# Most Relevant Spectral Bands Identification for Brain Cancer Detection Using Hyperspectral Imaging

**DOI:** 10.3390/s19245481

**Published:** 2019-12-12

**Authors:** Beatriz Martinez, Raquel Leon, Himar Fabelo, Samuel Ortega, Juan F. Piñeiro, Adam Szolna, Maria Hernandez, Carlos Espino, Aruma J. O’Shanahan, David Carrera, Sara Bisshopp, Coralia Sosa, Mariano Marquez, Rafael Camacho, Maria de la Luz Plaza, Jesus Morera, Gustavo M. Callico

**Affiliations:** 1 Institute for Applied Microelectronics (IUMA), University of Las Palmas de Gran Canaria (ULPGC), 35017 Las Palmas de Gran Canaria, Spain; slmartin@iuma.ulpgc.es (R.L.); hfabelo@iuma.ulpgc.es (H.F.); sortega@iuma.ulpgc.es (S.O.); gustavo@iuma.ulpgc.es (G.M.C.); 2 Department of Neurosurgery, University Hospital Doctor Negrin of Gran Canaria, 35010 Barranco de la Ballena s/n, Las Palmas de Gran Canaria, Spain; pinerbrains1@yahoo.es (J.F.P.); adamszolna@wp.pl (A.S.); hhdez.maria@gmail.com (M.H.); carlosespinopostigo@gmail.com (C.E.); aruosha@gmail.com (A.J.O.); david__carrera@hotmail.com (D.C.); sarabisshop@hotmail.com (S.B.); coralia.sosa@gmail.com (C.S.); marquezrdguez@yahoo.es (M.M.); jmormol@gobiernodecanarias.org (J.M.); 3 Department of Pathological Anatomy, University Hospital Doctor Negrin of Gran Canaria, 35010 Barranco de la Ballena s/n, Las Palmas de Gran Canaria, Spain; rcamgal@gobiernodecanarias.org (R.C.); mplaperb@gobiernodecanarias.org (M.d.l.L.P.)

**Keywords:** brain cancer, hyperspectral imaging, intraoperative imaging, feature selection, image-guided surgery, genetic algorithm, particle swarm optimization, ant colony optimization, support vector machine, machine learning

## Abstract

Hyperspectral imaging (HSI) is a non-ionizing and non-contact imaging technique capable of obtaining more information than conventional RGB (red green blue) imaging. In the medical field, HSI has commonly been investigated due to its great potential for diagnostic and surgical guidance purposes. However, the large amount of information provided by HSI normally contains redundant or non-relevant information, and it is extremely important to identify the most relevant wavelengths for a certain application in order to improve the accuracy of the predictions and reduce the execution time of the classification algorithm. Additionally, some wavelengths can contain noise and removing such bands can improve the classification stage. The work presented in this paper aims to identify such relevant spectral ranges in the visual-and-near-infrared (VNIR) region for an accurate detection of brain cancer using in vivo hyperspectral images. A methodology based on optimization algorithms has been proposed for this task, identifying the relevant wavelengths to achieve the best accuracy in the classification results obtained by a supervised classifier (support vector machines), and employing the lowest possible number of spectral bands. The results demonstrate that the proposed methodology based on the genetic algorithm optimization slightly improves the accuracy of the tumor identification in ~5%, using only 48 bands, with respect to the reference results obtained with 128 bands, offering the possibility of developing customized acquisition sensors that could provide real-time HS imaging. The most relevant spectral ranges found comprise between 440.5–465.96 nm, 498.71–509.62 nm, 556.91–575.1 nm, 593.29–615.12 nm, 636.94–666.05 nm, 698.79–731.53 nm and 884.32–902.51 nm.

## 1. Introduction

Globally, around 260,000 brain tumor cases are detected each year, with the main brain tumor type being detected the glioblastoma multiforme (GBM) that has the highest death rate (22%) [1]. This type of cancer leads to death in children under the age of 20, and also is one of the principal causes of death among 20- to 29-year-old males [2]. Surgery is one of the principal treatments alongside radiotherapy and chemotherapy [3]. During surgery, several image guidance tools, such as intra-operative neuro-navigation, intra-operative magnetic resonance imaging (iMRI) and fluorescent tumor markers, have been commonly used to assist in the identification of brain tumor boundaries. However, these technologies have several limitations, producing side effects in the patient or invalidating the patient-to-image mapping, reducing the effectiveness of using pre-operative images for intra-operative surgical guidance [4].

Hyperspectral imaging (HSI) is a technology that combines conventional imaging and spectroscopy to obtain simultaneously the spatial and the spectral information of an object [5]. Hyperspectral (HS) images provide abundant information that covers hundreds of spectral bands for each pixel of the image. Each pixel contains an almost continuous spectrum (radiance, reflectance or absorbance), acting as a fingerprint (the so called spectral signature) that can be used to characterize the chemical composition of that particular pixel [5]. One of the main advantages of this technique is that it uses non-ionizing light in a non-contact way, resulting in a non-invasive technology. For this reason, HSI is an emerging technique in the medical field and it has been researched in many different applications, such as oximetry of the retina [6,7,8], intestinal ischemia identification [9], histopathological tissue analysis [10,11,12,13], blood vessel visualization enhancement [14,15], estimation of the cholesterol levels [16], chronic cholecystitis detection [17], diabetic foot [18], etc. In particular, HSI has started to achieve promising results in the recent years with respect to cancer detection through the utilization of cutting-edge machine-learning algorithms [4,19,20,21]. Several types of cancer have been investigated using HSI including both in vivo and ex vivo tissue samples, such as gastric and colon cancer [22,23,24,25], breast cancer [26,27], head and neck cancer [28,29,30,31,32,33], and brain cancer [34,35,36], among others.

This imaging modality is mainly characterized by the curse of dimensionality, produced due to the high dimensionality of the data in contrast to the low number of available samples. This rich amount of data allows having more detailed information about the scene that is being captured. However, it also causes a large increase of the computing time required to process the data, containing normally redundant information [37]. For this reason, it is necessary to employ processing algorithms able to reduce the dimensionality of the HS data without losing the relevant information. This dimensional reduction process consists in the transformation of the data, characterized by their high dimensionality, into a significant representation of such data in a reduced dimension [38]. There are two main types of methods for dimensionality reduction: feature extraction [39] and feature selection [40]. Feature extraction algorithms are able to reduce, scale and rotate the original feature space of the HS data through a transformation matrix. This transformation optimizes a given criterion on the data so they can be formulated as a linear transformation that projects feature vectors on a transformed subspace defined by relevant directions. On the other hand, feature selection algorithms applied to HS images aim to find the optimal subset of bands in such images, performing several combinations of bands in a certain way until the best subset is found. This process reduces the dimensionality of the data by selecting the most discriminant bands of the dataset. Some of the most common algorithms for feature selection are optimization algorithms such as the Genetic Algorithm (GA) [41], Particle Swarm Optimization (PSO) [42], and Ant Colony Optimization (ACO) [43], among the most relevant.

The main goal of this work is to evaluate different band selection algorithms in order to identify the minimum number of wavelengths to sample in HS images using a supervised classifier that are necessary to process in vivo human brain HS data. This wavelength reduction will allow an accurate delineation of brain tumors during surgical procedures, obtaining similar results to the classification performed by using the original number of wavelengths. In this sense, a straightforward Support Vector Machine (SVM) classifier has been used instead of other more advanced ones to avoid hiding the band selection procedure effects. The use of feature selection algorithms was motivated by the goal of providing insights about the relevant spectral regions for this task, offering the possibility of reducing the number of spectral bands that the HS sensor has to capture. This will lead to a reduction of the acquisition system size and costs, as well as an acceleration of the execution time required by the processing algorithms. In this sense, the use of customized snapshot HS cameras coupled with a surgical microscope, could be considered to capture real-time HS data during brain surgery. This type of cameras can capture HS video imaging but with a reduced number of spectral channels. The surgical microscopic-based HS system will be the future replacement of the current macroscopic HS capturing system based on push-broom HS cameras, which requires at least 1 min to capture the entire HS cube, employed in the intraoperative HS brain cancer detection research [35]. Band reduction techniques will be crucial to allow real time acquisition using snapshot HS cameras.

## 2. Materials

This section describes the HSI instrumentation used to generate the in vivo HS brain cancer image database, as well as the proposed band selection-based methodologies. In addition, the evaluation methodology and the metrics employed to validate the results are presented.

### 2.1. Intraoperative Hyperspectral (HS) Acqusition System

In order to obtain the in vivo human brain cancer database, a customized intraoperative HS acquisition system was employed [35]. The acquisition system was composed of a VNIR (visible and near infra-red) push-broom camera (Hyperspec^®^ VNIR A-Series, Headwall Photonics Inc., Fitchburg, MA, USA), providing HS images in the spectral range from 400 to 1000 nm. The HS cubes were formed by 826 spectral bands, having a spectral resolution of 2–3 nm and a spatial resolution of 128.7 µm. Due to the push-broom nature of the HS camera, the sensor is a 2-D detector with a dimension of 1004 × 826 pixels, capturing the complete spectral dimensions and one spatial dimension of the scene at once. For this reason, the system requires a scanning platform in order to shift the field of view of the camera relative to the scene that is going to be captured in order to obtain the second spatial dimension. Considering the employed scanning platform, the maximum size of the HS image is 1004 × 1787 pixels and 826 spectral bands, covering an area of 129 × 230 mm with a pixel size of 128.7 µm. Furthermore, a specific illumination system able to emit cold light in the spectral range between 400 and 2200 nm has been coupled to the system. A 150 W QTH (quartz tungsten halogen) source light is attached to a cold light emitter via fiber optic cable that avoids the high temperatures produced by the lamp in the exposed brain surface.

### 2.2. In Vivo Human Brain Cancer Database

The in vivo human brain cancer database employed in this study is composed by 26 HS images obtained from 16 adult patients. Patients underwent craniotomy for resection of intra-axial brain tumor or another type of brain surgery during clinical practice at the University Hospital Doctor Negrin at Las Palmas de Gran Canaria (Spain). Eleven HS images of exposed tumor tissue were captured from eight different patients diagnosed with grade IV glioblastoma (GBM) tumor. The remaining patients were affected by other types of tumors or underwent a craniotomy for stroke or epilepsy treatment. From these patients, only normal brain tissue samples were recorded and employed in this study. The tumor samples different from GBM were not included in this study. Moreover, in the GBM cases where the tumor area was not able to be captured in optimal conditions, mainly due to the presence of extravasated blood or surgical serum, these images were included in the database but no tumor samples were employed. Finally, only GBM tumor samples belonging to six HS images from four different patients were employed. Written informed consent was obtained from all participant subjects and the study protocol and consent procedures were approved by the Comité Ético de Investigación Clínica-Comité de Ética en la Investigación (CEIC/CEI) of the University Hospital Doctor Negrin.

The following protocol was performed to acquire the data during the surgical procedures. After craniotomy and resection of the dura, the operating surgeon initially identified the approximate location of the normal brain and tumor (if applicable). At that point, sterilized rubber ring markers were located on those places and the HS images were recorded with markers in situ. Next, the tissue inside the markers were resected, and histopathological examination was performed for the final diagnosis. Depending on the location of the tumor, images were acquired at various stages of the operation. In the cases with superficial tumors, some images were obtained immediately after the dura was removed. In the cases of deep-lying tumors, images were captured during the actual tumor resection. More details about this procedure can be found in [35].

From the obtained HS cubes, a specific set of pixels was labeled using four different classes: tumor tissue, normal tissue, hypervascularized tissue (mainly blood vessels), and background. The background class involves other materials or substances presented in the surgical scenario but not relevant for the tumor resection procedure, such as skull bone, dura, skin or surgical materials. The labeling of the images was performed using a combination of pathology assessment and neurosurgical criteria using a semi-automatic tool based on the Spectral Angle Mapper (SAM) algorithm [44]. In this procedure, the operating surgeon employed the semi-automatic labeling tool for a supervised selection of a reference pixel in the HS image where the neurosurgeon was very confident that it belonged to a certain class. Then, the SAM was computed in the entire image with respect to the reference pixel and a threshold was manually established to identify the pixels with the most similar spectral properties to the selected one. Tumor pixels were labeled according to the histopathological diagnosis obtained from the biopsies performed in a certain area (indicated by the rubber ring markers) during surgery. Normal, hypervascularized and background pixels were labeled according to the neurosurgeon experience and knowledge. During this supervised labeling procedure, special attention was paid to avoid the inclusion of pixels in more than one class. On average, 6% of the pixels where labeled from each HS cube available in the database.

It is worth noting that the non-uniformity of the brain tissue produced the presence of specular reflections in the HS image of the captured scene. As described in the previous section, the acquisition system was based on a push-broom HS camera, equipped with a high-power illumination device connected to a linear cold-light emitter, thus avoiding interference of the environment illumination in the capturing process. The incident light beam emitted over the brain surface only illuminates the line captured by the HS camera, and both the camera and the light beam were shifted to capture the entire HS cube. The use of the required powerful illumination together with the non-uniformity of the brain surface, the inherent movement of the exposed brain and the movement of the HS scanning platform make extremely difficult to avoid specular reflections in the HS image. This challenging problem has been investigated in many applications, especially in works related with the analysis of in vivo and ex vivo head and neck cancer samples through HSI [45,46]. In our case, we excluded the use of these glare pixels for the quantitative processing of the HS data. During the supervised labeling procedure, glare pixels were avoided, i.e., glare pixels are not included in the labelled dataset. Hence, both the training and the quantitative classification of the data were not affected by the specular reflections. However, in the qualitative results based on classification maps where the entire HS cube is classified, the glare pixels were classified and we realized that such pixels were mostly identified as background. More information about the acquisition of the HS data and the generation of the labeled dataset can be found in [47].

**Figure 1:**
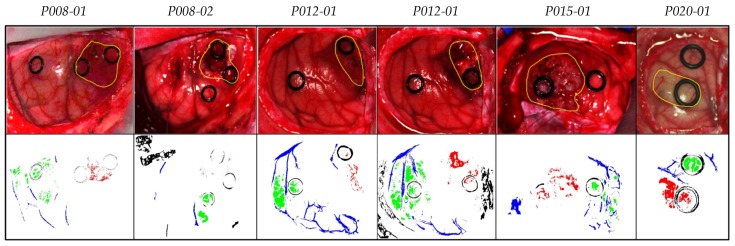
Synthetic RGB (Red, Green and Blue) images of HS test dataset with the tumor area surrounded in yellow (first row) and gold standard maps obtained with the semi-automatic labeling tool from the HS cube (second row). Normal, tumor, hypervascularized and background classes are represented in green, red, blue, and black color, respectively. White pixels correspond with non-labeled data.

Table 1 shows the number of labeled samples per class employed in this work, while Table S1 from the supplementary material details the patients and the number of samples per class and per image that were involved in the experiments according to the dataset published in [47]. As previously mentioned, from the eight patients affected by GBM tumor, only tumor pixels from four patients were labeled. In total, six HS images were labeled with four classes and were employed as a test dataset. Figure 1 shows the synthetic RGB (red green blue) images of the HS cubes with the tumor areas surrounded in yellow and the ground truth maps of each HS image employed for the test evaluation of the algorithms throughout a leave-one-patient-out cross-validation methodology. This cross-validation methodology was selected in order to perform an inter-patient validation and to avoid overfitting in the supervised classification model generation. Due to the low number of HS images with tumor pixels labeled, it was not possible to perform another evaluation approach based on training, validation and test data partition. In the ground truth maps the green, red, blue, and black pixels represents the normal, tumor, hypervascularized, and background labeled samples, respectively. The white pixels represent the pixels that have no class assigned, so it is not possible to perform a quantitative evaluation of such pixels. The classification of the entire HS cube is only evaluated in a qualitative way. The identification numbers of the test HS cubes correspond with those presented in [47].

**Figure 2:**
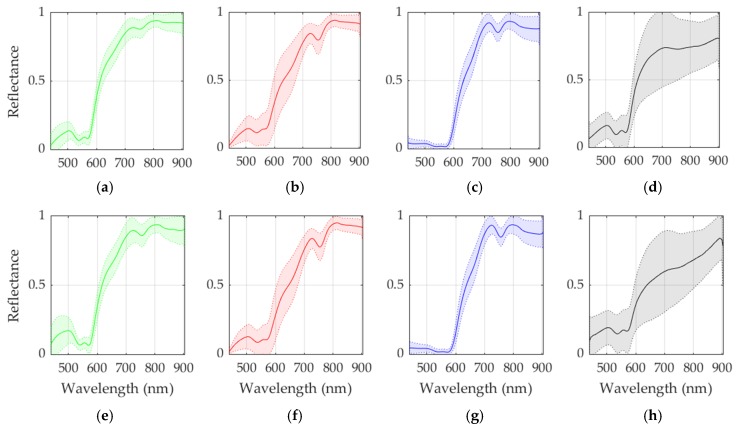
Average and standard deviation spectral signatures of the original and reduced dataset per classes. (**a**–**d**) Normal, tumor, hypervascularized and background spectral signatures respectively, extracted from the original training dataset. (**e**–**h**) Normal, tumor, hypervascularized and background spectral signatures respectively, extracted from the reduced training dataset.

Figure 2 shows the average and standard deviations of the spectral signatures available in both the original and the reduced training datasets. As can be seen, there are minimal differences in the average and standard deviation of the normal, tumor and hypervascularized classes while in the background class the differences are more noticeable. This is mainly produced due to this class involving several different materials that can be found in a neurosurgical scene, such as the skull bone, dura matter, gauzes with and without blood, plastic pins, etc. These materials have highly different spectral signatures which is evidenced by the high standard deviation obtained for this class.

From the point of view of the biological analysis, certain wavelength ranges have been associated to particular optical properties of cancer tissues [4]. The major spectral contribution of hemoglobin (Hb) is found in the range between 450 and 600 nm [48]. Particularly, deoxygenated Hb shows a single absorbance peak around 560 nm, while oxygenated Hb shows two equal absorbance peaks around 540 and 580 nm [49]. On the other hand, the region of the NIR spectrum from 700 to 900 nm corresponds with the scattering dominant optical properties of biological tissues, mainly composed of fat, lipids, collagen, and water [50]. Considering that the absorbance peaks are transformed to valleys in reflectance measurements, within the spectral signatures of the normal and tumor classes in Figure 2, these valleys in the range between 540 and 580 nm can be identified.

## 3. Methodology

The general methodology employed in this work to evaluate the results obtained was based on a SVM classifier [51], following a data partition consisting on a leave-one-patient-out cross-validation. This method performs an inter-patient classification where the samples of an independent patient are used for the test, while the training group includes all the patients’ samples except the ones to be tested. This process is repeated for each patient in the test database. The SVM classifier was selected in order to compare the results with previous published works [34,36,52]. However, although in the previous works deep learning approaches were evaluated, in this preliminary study only the SVM-based approaches were evaluated and compared, mainly because of the limited sample size. The SVM implementation was performed in the MATLAB^®^ R2019a (The MathWorks, Inc., Natick, MA, USA) environment using the LIBSVM package developed by Chang et al. [53]. Following this general methodology, three different processing frameworks have been proposed.

**Figure 3:**
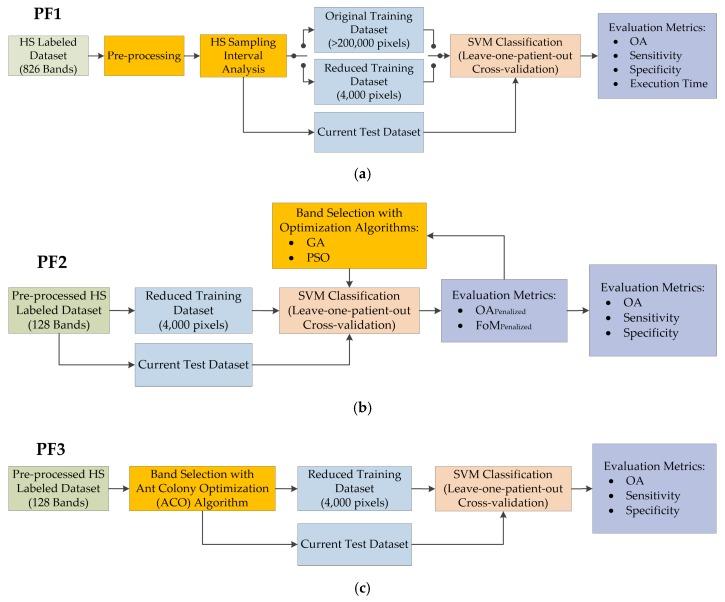
Different processing frameworks (PF) evaluated in this work. (**a**) *PF1* where the HS sampling interval analysis is performed with the original and the reduced training datasets independently. (**b**) *PF2* where the analysis of the *GA* and *PSO* optimization algorithms is performed using only the reduced training dataset and the optimal sampling interval selected in PF1. (**c**) *PF3* where the *ACO* algorithm is evaluated using the same training and input datasets as those employed in *PF2*. In these figures, green blocks represent the input data, orange blocks identify the main part of the proposed framework, blue blocks denote the train and test datasets employed for the supervised classification (pale brown blocks), and purple blocks represent the evaluation metrics employed.

The first processing framework (*PF1*) performs a sampling interval analysis of the HS data (composed by 826 bands) in order to evaluate the reduction of the number of bands in the HS images by modifying the sampling interval of the HS camera, i.e., decimating the bands to be employed in the classification process at certain steps. This procedure is intended to reduce the redundant information in the data due to the high dimensionality, allowing also a decrease in the execution time of the classification algorithm. In addition, in this processing framework, a training dataset reduction algorithm based on the K-means clustering algorithm is proposed with the goal of reducing the number of samples in the training dataset. By employing this method, the most relevant information is employed to train the SVM classifier, balancing the training samples for each class of the dataset and drastically reducing the training execution time. This time reduction obtained in the sampling interval analysis and the training dataset reduction will be crucial in the next processing frameworks, where the analysis of different optimization algorithms is performed. The block diagram of the *PF1* is shown in Figure 3a.

The second processing framework (*PF2*) has the goal of evaluating the *GA* and *PSO* optimization algorithms as band selection methods. In this framework, the suitable solutions obtained in the *PF1* are used and specific evaluation metrics are employed to iteratively find the best solution. Finally, general evaluation metrics for classification tasks are employed to obtain the final results for each case. Figure 3b shows the block diagram of this processing framework.

The third processing framework (*PF3*) evaluates the use of the *ACO* algorithm to find the most relevant bands. This algorithm works in a different way than the *GA* and *PSO*. The *ACO* algorithm sorts the different bands according to their importance and correlation between them. Thus, the iterative procedure is not required. In Figure 3c, the block diagram of this process is shown.

In next sections, each one of these proposed processing frameworks are explained in detail. To improve the readability of the rest of the manuscript, a list of the acronyms of each proposed method that will be named in the results section is presented in Appendix A in Table A1.

### 3.1. Processing Framework 1 (PF1): Sampling Interval Analysis and Training Dataset Reduction

The *PF1* (Figure 3a) aims to perform a sampling interval analysis of the HS raw data in order to reduce as much as possible the number of bands required to obtain accurate classification results, removing the redundant information provided by the high spectral resolution of the HS camera. In addition, in this processing framework a training dataset reduction algorithm is proposed with the goal of reducing the high training times of the SVM classifier. The best pre-processing chain obtained in this analysis, involving also the sampling interval selection, together with the optimal training dataset selection, will be employed in the next proposed processing frameworks.

#### 3.1.1. Data Pre-Processing

A pre-processing chain composed of three main steps was applied to the HS raw data in order to homogenize the spectral signatures of the dataset. The first stage in this chain is the radiometric calibration of the HS images, performed to avoid the interference of environmental illumination and the dark currents of the HS sensor. The raw data was calibrated using white and dark reference images following Equation (1), where $C_{i}$ is the calibrated image, $R_{i}$ is the raw image, and $W_{r}$ and $D_{r}$ are the white and dark reference images, respectively. The white reference image was captured from a material that reflects the 99% of the incoming radiation in the full spectral range considered in this work (Spectralon^®^ tile). This tile was placed at the same location where the patient’s head will be placed during the surgery, thus taking into account the real light condition. On the other hand, the dark reference image was acquired by keeping the camera shutter closed. This calibration procedure ensures the consistence of data and the reproducibility of the results independent of the operating room where the system is used.

(1) $$C_{i}=100\cdot \frac{R_{i}-D_{r}}{W_{r}-D_{r}}$$

The second stage consists in the application of a noise filter in order to reduce the high spectral noise generated by the camera sensor using a smooth filter. Except for the first case of the sampling interval analysis, presented in the next section, the preprocessing applied involves an extreme band-removing step before the noise filter with the goal of eliminating the bands with high noise in the first and last bands of the HS data produced with the low performance of the sensor in these bands. After this procedure, the operating bandwidth of the HS data is between 440 and 902 nm. Finally, in the third stage, the spectral signatures are normalized between zero and one in order to homogenize reflectance levels in each pixel of the HS image produced by the non-uniform surface of the brain. Equation (2) shows this process, where the normalized pixel ($P\prime_{i})$ is computed by subtracting the minimum reflectance value in a certain pixel ($P_{min}$) by the reflectance value of such pixel in a certain wavelength ($P_{i}$) and dividing it by the difference between the maximum and minimum reflectance values ($P_{max}-P_{min})$.

(2) $$P\prime_{i}=\frac{P_{i}-P_{min}}{P_{max}-P_{min}}$$

#### 3.1.2. Sampling Interval Analysis

In order to simulate the use of different HS cameras where a different number of spectral bands are captured covering the same spectral rage, the following methodology was employed to vary the spectral sampling interval of the HS data. The main goal of this analysis is to reduce the number of bands employed for the classification in this particular application and, consequently, the HS camera size and cost, as well the computational effort.

The spectral sampling interval is the distance between adjacent sampling points in the spectrum or spectral bands. This spectral sampling interval is calculated by Equation (3), where λmax−λmin is the difference between the maximum and minimum wavelength captured by the sensor, also named as the spectral range. The number of spectral bands have been reduced while the sampling interval increase in order to simulate diverse HS cameras that capture different number of bands.

(3) $$Sampling\;Interval\;\left(nm\right)=\frac{\lambda_{max}-\lambda_{min}}{number\;of\;bands}$$

Table 2 shows the different sampling interval values obtained for each number of spectral bands chosen. The original raw HS image captured by the sensor is composed by 826 bands, having 2–3 nm of spectral resolution and 0.73 nm of sampling interval. This HS camera covers the range between 400 and 1000 nm. In order to avoid the noise produced by the CCD (charge-coupled device) sensor in the extreme bands, they were removed, obtaining a final spectral range from 440 to 902 nm with 645 spectral bands and the same sampling interval. Using this HS cube as a reference, several sampling intervals were applied, reducing the number of bands and, in consequence, the size of the HS image. The original raw image size was higher than 1 GB and by reducing spectral bands from 826 to 8 the image size obtained was ~12 MB. These data were obtained from an average value of the HS test cubes, since the spatial dimension of each HS cube were different.

#### 3.1.3. Training Dataset Reduction

Supervised classifiers rely on the quality and amount of the labeled data to perform the training and create a generalized model to produce accurate results. However, in some cases, the labeled data can be unbalanced between the different classes and may contain redundant information, increasing the execution time of the training process and even worsening the performance of the classification results. Taking into account that the optimization algorithms used in this work have to perform an iterative training of the classifier in order to find the most relevant wavelengths for obtaining an accurate classification, it is beneficial to accelerate the training process.

**Figure 4:**
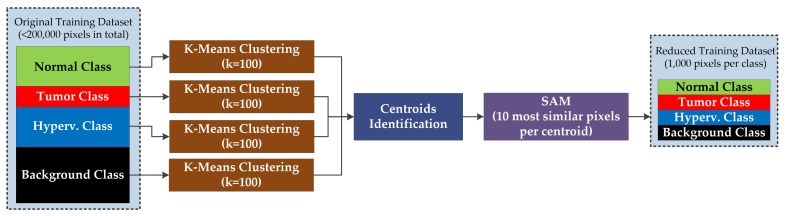
Block diagram of the training dataset reduction algorithm.

The methodology proposed in this section for optimizing the training dataset is based on the use of K-Means unsupervised clustering [54]. Figure 4 shows the block diagram of the proposed approach for reducing the training dataset. In this approach, the training dataset is separated in four groups that correspond with the different classes available in the labeled dataset: normal, tumor, hypervascularized and background. The total number of samples available in the entire dataset is 269,676 pixels, corresponding to 101,706 normal pixels, 11,054 tumor pixels, 38,784 hypervascularized pixels, and 118,132 background pixels (see Table 1). The K-Means clustering is applied independently to each group of labeled pixels in order to obtain 100 different clusters (K = 100) per group (400 clusters in total). Hence, 100 centroids that correspond to a certain class are obtained. In order to reduce the original training dataset, such centroids are employed to identify the most representative pixels of each class by using the SAM algorithm [44]. For each centroid, only the 10 most similar pixels are selected, having a total of 1000 pixels per class (100 centroids × 10 pixels). Thus, the reduced dataset is intended to avoid the inclusion of redundant information in the training of the supervised classifier. At the end, the reduced training dataset will be composed of 4000 pixels (after applying the K-Means four times independently) being the most representative pixels of the original dataset and obtaining a balanced dataset among the different classes. It is worth noticing that this procedure is executed within the leave-one-patient-out cross-validation methodology. Thus, the labeled pixels of the current test patient will not be included in the original dataset employed for the reduction process.

### 3.2. PF2: Band Selection Using Genetic Algorithm (GA) and Particle Swarm Optimization (PSO)

In this processing framework (Figure 3b), the use of the *GA* and *PSO* for the selection of the most representative wavelengths of the HS data for this particular application is proposed. The optimization algorithms are aimed to find the best combination of elements from an initial set of available elements. Normally, these algorithms are focused on reaching the global minimum of the function to be analyzed. The reduced training labeled dataset evaluated in *PF1* was employed to reduce the extremely high execution time of the SVM training procedure, due to the iterative nature of the optimization algorithms. The procedure is as follows. First, the training data are employed in the optimization algorithm, where the initial bands to be used for the classification are randomly selected. After this band selection procedure, a classification model is generated and evaluated with the test dataset, obtaining a classification result that is assessed using three custom metrics independently (*OA_Penalized_, FoM* and *FoM_Penalized_*). These metrics will be explained in detail later on in Section 3.5. After computing one of the custom metrics, its value is stored and then the procedure is iteratively executed using other bands selected by the optimization algorithm. The algorithm is executed until performs all possible combinations, returning the best metric value, or when after a high number of iterations, the metric value remains constant. Once the algorithm finishes its execution, it returns the identification of the optimal bands to obtain the best classification result.

#### 3.2.1. Genetic Algorithm (GA)

The *GA* is an optimization algorithm that mimics the process of natural selection [41]. *GA* tries to find the optimal solution (usually the global minimum) of the function to be studied. The main advantage of this algorithm is its great ability to work with a large number of variables [55,56]. The objective of this algorithm is to optimize a series of parameters (called genes) that will then be concatenated with each other, when necessary, to provide the best results (called chromosomes). In order to find these most important parameters, it is necessary to generate populations in a random manner, whose size is chosen by the user. This population allows the performance of the algorithm to be improved. Once these parameters are defined, the *GA* must perform the following steps:

(1) **Initialization**: In this step, the selection of the population is performed in a random way.
(2) **Evaluation**: The goal is to study the results obtained from the initial population (*parents*) and each of the descendant generations (*children*).
(3) **Selection**: This point is responsible for keeping the best result obtained during the evaluation process.
(4) **Recombination**: In this step, the combination of the different initial contributions (*parents*) for the creation of better solutions (*children*) is performed. This crossing is performed by dividing the populations in two (or more) parts and exchanging part of those populations with each other.
(5) **Mutation**: This technique is performed in the same way as in the recombination step. However, instead of exchanging parts of the populations among themselves, a single value of each of the populations is modified.
(6) **Replacement**: After performing the recombination and mutation steps, these generations (*children*) replace the initial populations (*parents*).

Steps 2 to 6 are repeated as many times as necessary until the best solution is found [41].

#### 3.2.2. Particle Swarm Optimization (PSO) Algorithm

The PSO algorithm is a stochastic technique based on the behavior of bee swarms [42]. This algorithm exhibits great effectiveness in multidimensional optimization problems. This methodology is based on the survival of some living beings (specifically the bees). As well as the genetic algorithm, PSO is initialized with a random initial population. However, in this case, each possible solution, known as a particle, has also been assigned a random velocity [42]. Each particle updates and stores the best position found so far (pbest) and also stores and updates the best position of the rest of the swarm (gbest). To represent the velocity update, the algorithm uses Equation (4), where $v_{i}$ is the velocity vector, $x_{i}$ is the position vector, α is the weight of the particle that controls the recognition of the place, $c_{1}$ and $c_{2}$ are the acceleration constants of the particles (usually take a value of 2 by default), and rand is a random number between 0 and 1.

(4) $$v_{i}\left(t+1\right)=\alpha v_{i}+c_{1}\cdot rand\cdot \left(pbest\left(t\right)-x_{i}\left(t\right)\right)+c_{2}\cdot rand\cdot \left(gbest\left(t\right)-x_{i}\left(t\right)\right)$$

Once the parameters that conform the algorithm are obtained, the swarm is generated by means of the following steps:

(1) **Initialization**: This step initializes a random population with different positions and velocities.
(2) **Selection**: In this step, each particle evaluates the best location found and the best position found by the rest of the swarm.
(3) **Evaluation**: Here, a comparison of all the results and selection of the *pbest* is performed. The same process is applied to find the best *gbest*.
(4) **Replacement**: In this last step, the new results replace the initial population and the process is repeated up to a maximum number of generations established by the user or until the solution converges.

### 3.3. PF3: Band Selection Using Ant Colony Optimization (ACO)

The *PF3* (Figure 3c) has the goal of selecting the optimal bands employing directly the *ACO* algorithm to the train dataset. In this case, the algorithm restructures the spectral bands of the HS data in order to allocate the most relevant bands, based on endmember extraction [57] (selection of pure spectra signature of the different materials) in the first positions and the least important ones at the end. After this band reorganizing procedure, a classification model was generated with the SVM classifier and evaluated with the standard evaluation metrics (accuracy, sensitivity and specificity). This process was generated five times, evaluating the 20, 40, 60, 80 and 100 most relevant bands obtained with the *ACO* algorithm.

#### Ant Colony Optimization (ACO) Algorithm

The *ACO* algorithm is based on a metaheuristic procedure, which aims to obtain acceptable solutions in problems of combinatorial optimizations in a reasonable computational time [43]. As the name suggests, this algorithm is based on the composition of the ant colonies. The ants, when searching for food, separate and begin to make trips in a random way. Once an ant gathers food, while carrying the food to the nest, it expels pheromones along the way. Depending on the quality or quantity of the food found, the amount of pheromones will vary. On the other hand, the evaporation of the pheromones causes the pheromones to disappear, so that, if these routes are not reinforced, they end up disappearing. This process is repeated until the best possible route is found.

Taking into account this selection process, the algorithm is characterized by having a main component, the *pheromone* model. This model is a parameterized probabilistic model, which consists of a vector of parameters that indicates the trajectory followed by *pheromones*. These values are updated until the minimum value of the problem is reached.

### 3.4. Coincident Bands Evaluation Methodology

When the optimization algorithms employed in *PF2* and *PF3* selected the bands, different bands were identified for each test image. Thus, the six HS test images were evaluated employing the same selected bands, i.e., using the coincident and non-coincident selected bands in each test image obtained in the leave-one-patient-out cross-validation for the SVM training and classification. The procedure was as follows:

- **First level (*L1*):** the coincident and non-coincident bands from all the test images were used to generate and evaluate the results.
- **Second level (*L2*):** the coincident bands repeated in at least two test images were used to generate and evaluate the results.
- **Third level (*L3***): the coincident bands repeated in at least three test images were used to generate and evaluate the results.

This process was repeated until reaching the possible six coincidences. However, in our case, a maximum of three levels of coincidence were obtained.

### 3.5. Evaluation Metrics

The validation of the proposed algorithm was performed using inter-patient classification, following a leave-one-patient-out cross-validation. Overall accuracy (OA), sensitivity and specificity metrics were calculated to measure the performance of the different approaches. OA is defined by Equation (5), where TP is true positives, TN is true negatives, P is positives, and N is negatives. Sensitivity and specificity are defined in Equations (6) and (7), respectively, where FN is false negatives, and FP is false positives. In addition, the Matthews correlation coefficient (MCC) was employed to evaluate the different approaches (Equation (8)). This metric is mainly used to analyze classifiers that work with unbalanced data, which computes the correlation coefficient between the observed and the predicted values [58]. MCC has a value range between [−1, 1], where −1 represents a completely wrong prediction and 1 indicates a completely correct prediction. For comparison purposes with other metrics presented in this work, the MCC metric has been normalized within the [0, 1] range applying Equation (9).

(5) $$OA=\frac{TP+TN}{P+N}$$

(6) $$Sensitivity=\frac{TP}{TP+FN}$$

(7) $$OA=\frac{TP+TN}{P+N}$$

(8) $$MCC=\frac{TP\cdot TN-FP\cdot FN}{\sqrt{\left(TP+FP\right)\cdot \left(TP+FN\right)\cdot \left(TN+FP\right)\cdot \left(TN+FN\right)}}$$

(9) $$MCC\prime =\frac{MCC+1}{2}$$

Classification maps are another evaluation metric commonly used in HSI. This evaluation metric allows users to visually identify where each of the different classes are located. This metric is employed to visually evaluate the classification results obtained when the entire HS cube is processed, including labeled and non-labeled pixels. After performing the classification of the HS cube, a certain color is assigned to each class. This process allows mainly evaluating the results obtained in the prediction of non-labeled pixels. The colors that are represented in the classification map are the following: green was assigned to the first class (healthy tissue); red was assigned the second class (tumor tissue); blue was assigned to the third class (hypervascularized tissue); and black was assigned to the fourth class (background).

In addition to the standard OA, an additional metric has been proposed for the identification of the best results obtained with the optimization algorithms but taking into account also the number of selected bands. This $OA_{Penalized}$ is based on the OA presented in Equation (5) but including a penalty in the case that a high number of bands is used. Equation (10) presents the mathematical expression to compute this $OA_{Penalized}$, where λ is the number of bands selected by the algorithm and $\lambda_{max}$ is the total number of bands in the original dataset.

The specific Figure of Merit (*FoM*) employed to obtain the most relevant bands with the optimization algorithms in the *PF2* has the goal of finding the most balanced accuracy results for each class, as observed in Equation (12), where n is the number of classes, i and j are the indexes of the classes that are being calculated. The mathematical expression of the $ACC_{perClass}$ in a multiclass classification is obtained by dividing the total number of successful results (TP) for a particular class by the total population of this class (TP+FN). This expression is equal to the sensitivity of a certain class in a multiclass classification problem. Equation (11) shows the mathematical expression of the $ACC_{perClass}$.

In addition to the previously presented *FoM*, another metric has been proposed for the identification of the best results obtained with the optimization algorithms but taking into account also the number of selected bands. This $FoM_{Penalized}$ is based on the FoM presented in Equation (12) but including a penalty in the case that a high number of bands is used. Equation (13) presents the mathematical expression to compute this $FoM_{Penalization}$, where λ is the number of bands selected by the algorithm and $\lambda_{max}$ is the total number of bands in the original dataset.

(10) $$OA_{Penalized}=1-\frac{OA}{1+\frac{\lambda }{\lambda_{max}}}$$

(11) $$ACC_{perClass}=\frac{TP}{TP+FN}$$

(12) $$FoM=\frac{1}{2}\cdot \left(\overset{n}{\underset{\begin{array}{c} i,j\\ i<j \end{array}}{\sum }}\frac{ACC_{i}+ACC_{j}}{\left|ACC_{i}-ACC_{j}\right|+1}\right)\cdot {\left(\begin{array}{c} n\\ 2 \end{array}\right)}^{-1}$$

(13) $$FoM_{Penalized}=1-\frac{FoM}{1+\frac{\lambda }{\lambda_{max}}}$$

## 4. Experimental Results and Discussion

This section will present the results obtained in the three proposed processing frameworks, as well as the overall discussion of the results. Table A1 in the Appendix A shows the acronym list of each proposed method named in the next sections in order to help the reader to follow the experimental results explanation.

### 4.1. Sampling Interval Analysis (PF1)

The *PF1* has the goal of performing a comparison between the use of different numbers of spectral bands in the HS database, modifying the sampling interval of the spectral data in order to simulate the use of different HS cameras and reducing the size of the database. This will lead to a reduction of the execution time of the processing algorithm. In addition, as shown in Figure 3a, the *PF1* was evaluated using two different training datasets for the SVM model generation: the original and the reduced training dataset.

**Figure 5:**
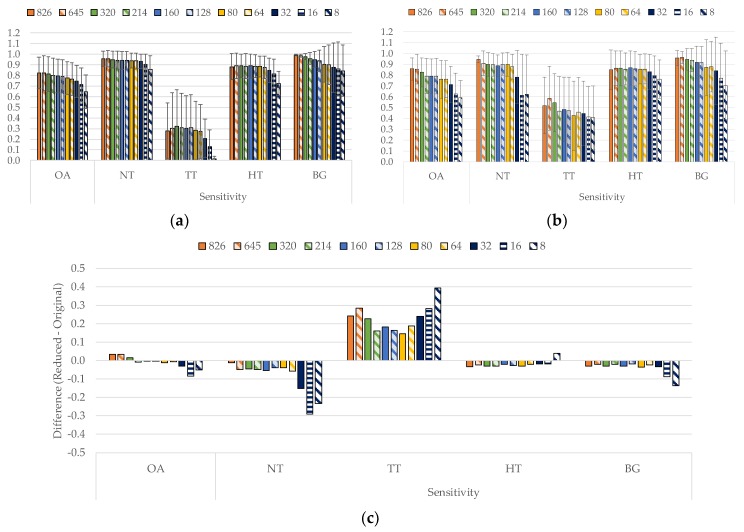
Average and standard deviation results of the leave-one-patient-out cross-validation for each band reduction. (**a**) Using the original training dataset. (**b**) Using the reduced training dataset. (**c**) Difference of the results computed using the reduced dataset respect to the original dataset.

Figure 5a shows the classification results obtained for each sampling interval (different number of bands), training the SVM algorithm with the original dataset, while Figure 5b shows the results using the reduced training dataset. It can be observed in both figures that the overall accuracy of the classifier decreases as the number of bands is reduced. However, the sensitivity values for each class are quite similar from 826 to 64 bands. When the number of bands is lower than 64, the sensitivity values drop drastically. Respect to the standard deviation, the behavior obtained in both datasets are similar. It can be observed that for the OA, normal sensitivity and hypervascularized sensitivity, the results remain stable as the number of bands are reduced. In the case of tumor sensitivity, the standard deviation is higher than in the other cases. This behavior is caused by one of the HS test images (*P020-01*) presenting problems in the classification and not being able to correctly identify any of the tumor pixels. In the background class, as the number of bands decrease, the standard deviation increases. On the other hand, as it can be seen in Figure S1 of the supplementary material, the specificity results are very similar in both cases being higher than 80% in general. In addition, Figure S2 of the supplementary material shows the results of the normalized MCC metric for the original and reduced training datasets, which takes into account the unbalance of the test labeled dataset. As can be seen, the obtained results in all the classes are similar except for the tumor tissue class, which improve an average of ~5% when the reduced training dataset is employed.

Figure 5c shows the differences in the results between the reduced and original training dataset. As it can be seen, the reduced dataset provides better accuracy results in the tumor class respect to the original dataset, reaching an average increment of ~20%. Since the main goal of this work is to accurately identify the tumor pixels, this increment provides a significant improvement on this goal. It is worth noticing that the test dataset was not reduced in the number of samples, only the training dataset was reduced.

**Figure 6:**
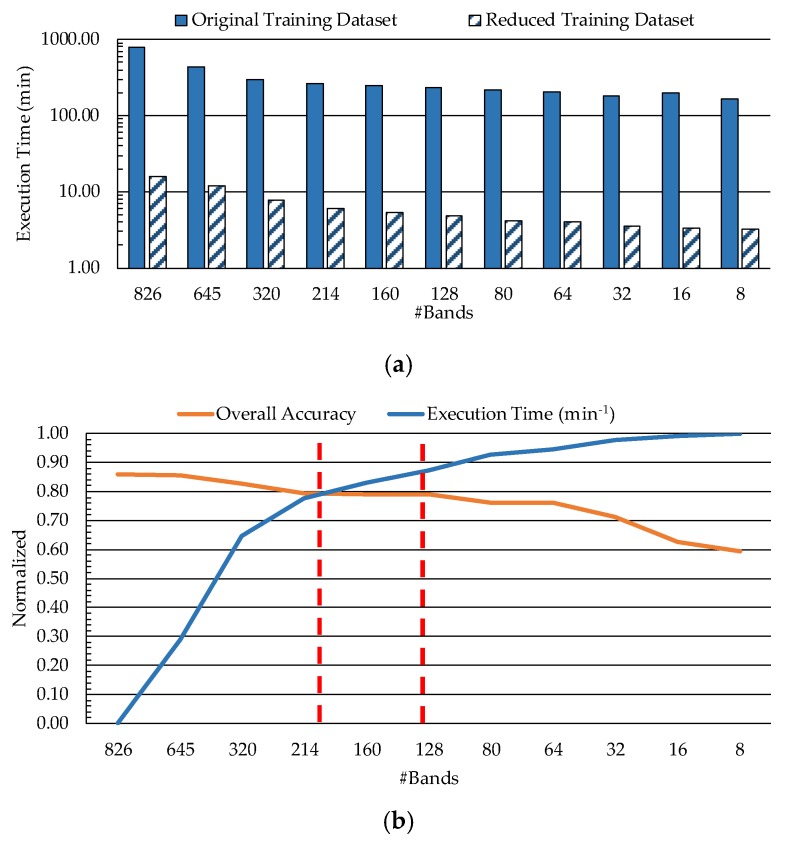
(**a**) Band reduction execution time for original and reduced training datasets (representation in logarithmic scale). (**b**) Overall accuracy and inverse normalized execution time achieved using the reduced training dataset with respect to the number of bands.

On the other hand, the image size is directly related to the execution time of the processing algorithm. Figure 6a shows the execution time of the SVM training and classification processes computed by using the MATLAB^®^ programming environment together with the LIBSVM package. This figure presents the execution time results (expressed in minutes) for both training schemes (original training dataset, and reduced training dataset). The times depicted in such a table include both the time required to train the model for one leave-one-patient-out cross-validation iteration, and the time needed to classify the correspondent patient data. In order to compare the results, a logarithmic scale was used. On one side, as the number of bands decreases, the execution time also decreases, being practically stable from 128 to 8 bands in both cases. On the other side, it is clear that the use of the reduced training dataset offers a significant execution time reduction. For example, using 826 bands, the execution time for the original training dataset is ~778 min, while for the reduced dataset it is ~16 min, obtaining a speedup factor of ~48×. Taking this into account, the reduced training dataset was selected for the next experiments.

In order to select a good trade-off sampling interval, which provides a reduction on the execution time of the algorithm while keeping high discrimination, a relation between these two metrics was performed. Figure 6b shows the relation between the accuracy and the inverse normalized execution time depending on the number of bands employed in the HS dataset, ranging from 826 to 8 bands. The analysis of the overall accuracy shows that when all the bands are used the overall accuracy is high, but the execution time is also very high. However, when only a few bands are used the overall accuracy decreases more than 20%, but the execution time is quite low. In this sense, the suitable range that provides a good compromise between the execution time and the overall accuracy is found between the 214 and 128 bands (dashed red lines in Figure 6b). In this range, the accuracy results are stabilized in the value of 80% and the execution times are acceptable. For this reason, the number of bands selected to conform the HS dataset in the next experiments was 128 (lowest number of bands with the same overall accuracy), involving a sampling interval of 3.61 nm.

The use of the reduced training dataset together with the selection of 128 spectral bands will ensure that we reduce the execution time (mainly in the SVM training process), allowing us to perform the band selection using the optimization algorithms in the next processing framework (PF2). This will avoid large processing times during the huge number of iterations required by the optimization algorithms until reaching the optimal solution.

### 4.2. Band Selection Using Optimization Algorithms (PF2 and PF3)

The *PF2* and *PF3* aim to use optimization algorithms in order to find the most relevant bands able to perform an accurate classification of the brain tumors, using the lower possible number of features. The evaluation of both processing frameworks was performed using the reduced training dataset, and a data partition scheme following a leave-one-patient-out cross-validation to create the SVM model and evaluate the results (see Figure 3b,c). In these processing frameworks, the six test HS images were evaluated with different optimization algorithms to find out which offers the best results. In addition, in case of *GA* and *PSO* algorithms (PF2), two different metrics were employed to evaluate the selected bands: $OA_{Penalized}$ (OA_P) and $FoM_{Penalized}$ (FoM_P). In summary, the band selection techniques evaluated were: *GA* using $OA_{Penalized}$ (PF2-GA-OA_P); *PSO* using $OA_{Penalized}$ (PF2-PSO-OA_P); *GA* using $FoM_{Penalized}$ (PF2-GA-FoM_P); *PSO* using $FoM_{Penalized}$ (PF2-PSO-FoM_P) and *ACO* using 60 bands (PF3-ACO-60). Furthermore, all the results were compared with the reference values obtained with the *PF1* using 128 bands (PF1-128).

**Figure 7:**
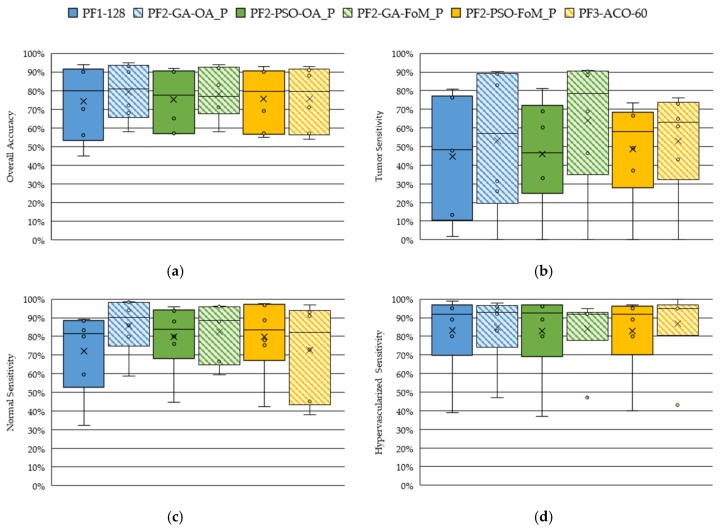
Boxplot results of the leave-one-patient-out cross-validation obtained for each processing framework. (**a**) Overall accuracy. (**b**) Tumor tissue sensitivity. (**c**) Normal tissue sensitivity. (**d**) Hypervascularized tissue sensitivity.

**Figure 8:**
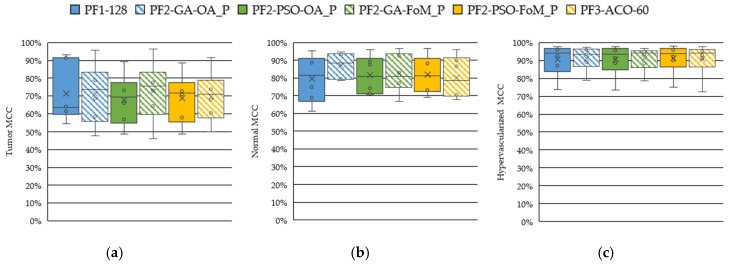
Boxplot results of the Matthews correlation coefficient (MCC) metric using the leave-one-patient-out cross-validation obtained for each processing framework. (**a**) Tumor tissue. (**b**) Normal tissue. (**c**) Hypervascularized tissue.

Figure 7 shows the boxplot results of the OA and the normal, tumor and hypervascularized tissue sensibilities obtained after the evaluation of the *GA*, *PSO* and *ACO* algorithms. The *ACO* algorithm was evaluated selecting different numbers of bands (20, 40, 60, 80 and 100), but only the results obtained with 60 bands have been reported because they were found to be the most competitive ones. Figures S3 and S4 in the supplementary material present the detailed results obtained with the *ACO* algorithm. Figure 7a shows the OA results of all the techniques, where it should be noted that the median values are around 80%, offering the PF2-GA-OA_P the best result. However, the results obtained in the tumor sensitivity boxplot (Figure 7b) shows that the technique that uses the *GA* algorithm with the $FoM_{Penalized}$ metric (PF2-GA-FoM_P) provides the best results, achieving a tumor sensitivity median of ~79%. This represents an increment of ~21% with respect to the PF2-GA-OA_P method. On the other hand, in Figure 7c, it can be seen that the results of the normal tissue sensitivity are similar in both cases (PF2-GA-OA_P and PF2-GA-FoM_P), with a median value of 89% and 90%, respectively. The same behavior can be observed in the hypervascularized tissue sensitivity results (Figure 7d). As can be observed in Figure 8, the MCC metric, which takes into account the unbalance of the test labeled dataset, shows the same behavior as the other metrics. Thus, the PF2-GA-FoM_P is the method that provides on average the best results. This is especially highlighted in the tumor class results.

**Figure 9:**
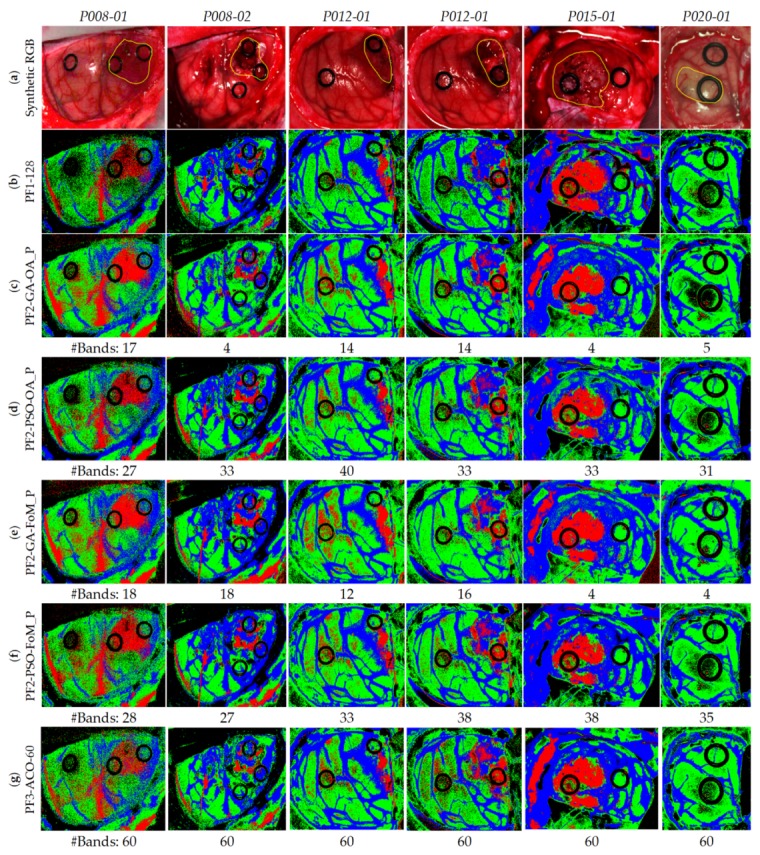
Classifications maps of the test database. (**a**) Synthetic RGB images with a yellow line identifying the tumor area. (**b**) Reference results using 128 bands. (**c**) Results of the *GA* algorithm using $OA_{Penalized}$. (**d**) Results of the *PSO* algorithm using $OA_{Penalized}$. (**e**) Results of the *GA* algorithm using $FoM_{Penalized}$. (**f**) Results of the *PSO* algorithm using $FoM_{Penalized}$. (**g**) Results of the *ACO* algorithm using the 60 bands per image.

Figure 9 illustrates the qualitative results represented in the classification maps obtained for each method. These maps allow visualization of the identification of the different structures for each class found in the complete HS cube, i.e., it is possible to visually evaluate the results obtained in the non-labeled pixels of the HS test cubes. In addition, this figure indicates the number of bands selected with each method for each HS test cube. Figure 9a shows the synthetic RGB images for each HS test cube, indicating the location of the tumor area surrounded by a yellow line, while Figure 9b shows the classification results obtained with the reference method (PF1-128).

Considering these images as reference, it is observed that the results in all cases are very similar. Nonetheless, when analyzing the images one-by-one, in the case of the first image, *P008-01*, it is observed that using either PF2-PSO-OA_P (Figure 9d) or PF3-ACO-60 (Figure 9g) the results are more accurate with fewer false positives. The best case that visualizes the *P008-02* image is the PF2-GA-FoM_P (Figure 9e), since it is observed a higher number of tumor pixels in the area of the tumor. As for the *P012-01*, all the techniques visualize the tumor area correctly, but the PF1-128 (Figure 9b) is the one that shows fewer false positives pixels. In the case of *P012-02*, the best technique is the PF2-GA-FoM_P (Figure 9e) due to it shows less false positive pixels. With respect to the *P015-01* image, it is observed that using PF2-GA-OA_P (Figure 9d) and PF2-GA-FoM_P (Figure 9e) the tumor area is clearly identified, although they have a small group of false positives in the upper left image due to some extravasated blood out of the parenchymal area. Finally, image *P020-01* offers practically the same result in all cases without a successful identification of the tumor area, even in the reference results. Regarding the number of bands selected to perform the classification, the PF2-GA-OA_P and PF2-GA-FoM_P are the methods that achieved the less number of bands for each HS test image, being lower than 18 bands in all the cases.

After conducting a thorough analysis of the results obtained, it was observed that the best technique, which provided the best balance between qualitative and quantitative results, is the PF2-GA-FoM_P. Quantitatively, the GA-FoM_P provided the best average OA value of 78% (improving ~4% with respect to the reference results with 128 bands) and the best median tumor sensitivity value of 79%, which represents an increment of ~21% with respect to the best solution provided with the other optimization approaches. Moreover, the GA-FoM_P increases the tumor sensitivity value in 30% with respect to the reference results.

### 4.3. Coincident Bands Evaluation of the GA Algorithm with Figure of Merit (FoM_Penalized_)

Taking into account the decision made in the previous section, the next step is the evaluation of the HS test images using the coincident bands in order to generate a general SVM model that can provide accurate results for all the HS test images. In this sense, the *PF2* using the *GA* algorithm and the $FoM_{Penalized}$ metric was evaluated by using the coincident and non-coincident bands selected during the cross-validation method over the six HS test images.

**Figure 10:**
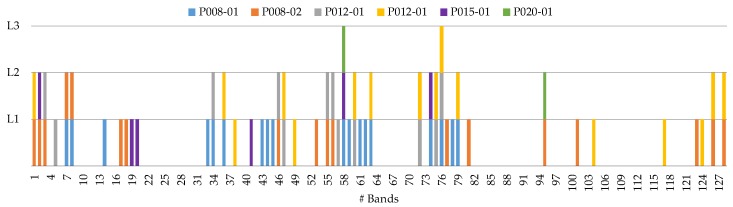
Graphical representation of the coincident and non-coincident bands obtained with the PF2-GA-FoM_P method (Processing Framework 2 using Genetic Algorithm and the Figure of Merit Penalized evaluation metric) for the three different levels.

The procedure followed for this evaluation consists of three levels. The index i, in Li, indicates the number of HS test images where the bands are common between all the test set. Figure 10 identifies the bands that were selected by the PF2-GA-FoM_P for each HS test image and the coincident bands between them. The number of bands for *L1*, *L2* and *L3* are 48, 22 and 2, respectively.

Table 3 shows the quantitative results obtained for the evaluation of the different levels. These results are the average and standard deviation of the six HS test images. In terms of OA, it is observed that the best result was obtained in *L1*, with 77.9%, followed closely by *L2* with 77.0%. However, *L3* worsens notably the results, achieving only 54%, mainly because of the low number of bands employed for the generation of the SVM model. With respect to the sensitivity results, *L1* and *L2* remain practically the same for all classes, being *L2* more accurate in the tumor tissue class. Nevertheless, *L3* worsens, especially in the normal and hypervascularized tissue classes. Regarding the specificity, it follows the same trend as in sensitivity, having *L1* and *L2* similar results and *L3* bears off from these results in the normal and tumor tissue classes.

**Figure 11:**
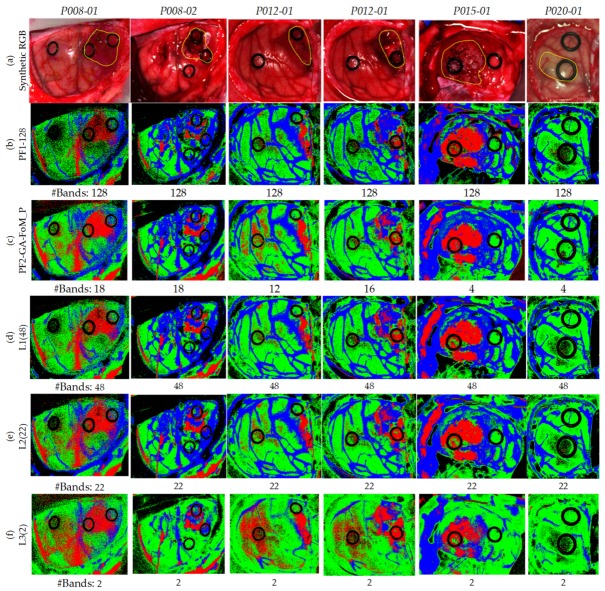
Maps of the test database using the different coincident levels. (**a**) Synthetic RGB images with a yellow line identifying the tumor area. (**b**) Reference results using 128 bands. (**c**) Results of the PF2-GA-FoM_P using the specific wavelengths identified for each HS test image. (**d**–**f**) Results of the PF2-GA-FoM_P in *L1*, *L2* and *L3*, respectively.

On the other hand, Figure 11 shows the qualitative results of each HS test image for the different levels, indicating below each classification map the number of bands employed to generate the classification model. Figure 11a shows the synthetic RGB images of each HS test image where the tumor area has been surrounded by a yellow line. Figure 11b shows the reference results obtained without applying the optimization methodology, so the 128 bands were employed. Figure 11c presents the classification results generated using the best methodology (PF2-GA-FoM_P) selected in the previous section, and employing the specific wavelengths obtained for each HS test image independently. Figure 11d–f show the classification results obtained using the *L1*, *L2*, and *L3* levels, respectively. In these results it is observed that *L3* (Figure 11f) provides several false positives in all the classes. For example, in the *P012-01* and *P012-02* images, a large number of tumor pixels (left side of the image) are presented in the normal tissue parenchymal area that are out of the surrounded yellow line presented in Figure 11a. Regarding *L1* (Figure 11d) and *L2* (Figure 11e), the results are very similar between them, with the only difference in the *P012-01* image which shows more false positive pixels in the tumor class in *L2* than in *L1*. Both quantitative and qualitative results obtained in *L3* show that the two selected wavelengths are not representative enough to generalize a classification model that offers accurate results for all the HS test images compared to *L1* and *L2*. By contrast, it is worth noticing that the results obtained in *L1* using only 48 bands are very similar and even better in some cases with respect to the results obtained with the reference method employing 128 bands (Figure 11b).

**Figure 12:**
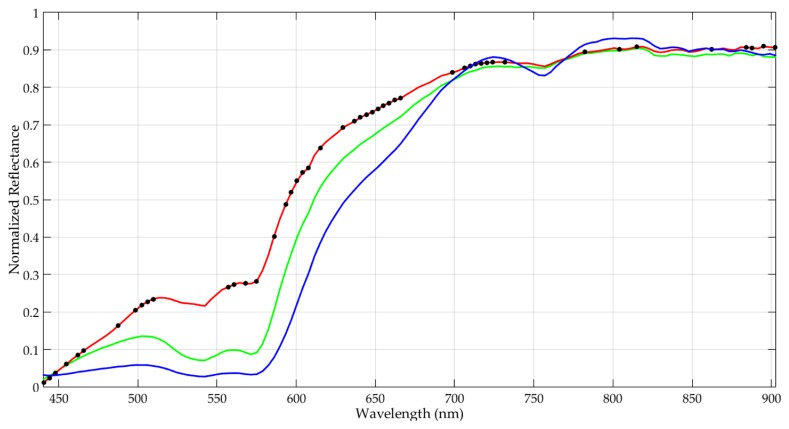
Example of the normalized average spectral signatures of the tumor (red), normal (green) and hypervascularized (blue) tissue classes. The black spots over the tumor spectral signature represent the 48 selected bands using the *GA* algorithm with *FoM_Penalized_* and the *L1* coincidence level.

Taking into account the quantitative and qualitative results obtained in these experiments, it has been concluded that the *L1* method provides the best accuracy results using only 48 bands. These selected bands represent the following spectral ranges: 440.5–465.96 nm, 498.71–509.62 nm, 556.91–575.1 nm, 593.29–615.12 nm, 636.94–666.05 nm, 698.79–731.53 nm and 884.32–902.51 nm. Figure 12 graphically represents the identification of the selected bands over an example of tumor, normal and hypervascularized spectral signatures. In addition, Table S2 of the supplementary material details the specific 48 wavelengths selected.

## 5. Conclusions

Hyperspectral images are able to capture a large number of spectral bands per pixel, conforming the so-called spectral signature. This type of images has high amount of information acquired by the sensors. Depending on the HS camera type, the HS images can be composed of thousands of spectral channels (involving large sizes in the range of gigabytes) and their processing requires high-performance computing in order to reduce the processing time as much as possible. In addition, the number of bands captured by the sensor normally implies different camera sizes and different acquisition methodologies, which in some cases are difficult to employ in certain applications. Thus, the large amount of data is one of the main challenges of HSI.

The work presented in this paper had the goal of analyzing the use of different sampling intervals to reduce the number of bands employed in the HS data. This led to accurate classification results with a reduced processing time being obtained and a possible future use for a reduced-size HS camera. Furthermore, a methodology to optimize the training dataset, employed to generate the SVM model, was proposed. This methodology offered a reduced training processing time and even achieved more accurate classification results due to the redundant information elimination and noise reduction. The reduced processing time for training is extremely important in the next steps of the work, aiming to evaluate different optimization algorithms (*GA*, *PSO* and *ACO*) for the selection of the most relevant bands in the delineation and identification of brain tumors.

The employed VNIR HS database was composed of 26 HS images of the in vivo human brain obtained during neurosurgical procedures. For each image, a certain number of pixels were labeled by the experts in four different classes (normal tissue, tumor tissue, hypervascularized tissue and background) in order to create a labeled dataset that was employed to generate and evaluate a SVM classification model. A leave-one-patient-out cross-validation methodology was followed using 6 HS test images of exposed brain from four different patients affected by GBM tumors pathologically confirmed.

Different processing frameworks were defined during the development of this work. The *PF1* demonstrated that the use of a sampling interval of 3.61 nm (128 bands, ~200 MB) instead of 0.73 nm (826 bands, ~1300 MB), together with the employment of the reduced training dataset (4000 pixels vs. ~200,000 pixels), provides quite an excellent compromise between the execution time and the accuracy of the results. Specifically, the speedup factor achieved in the execution time employing the reduced training dataset was ~48× with respect to the reference results (826 bands). Taking into account these results, the next step had the goal of identifying the most representative bands for each HS test image with different optimization algorithms (*PF2* and *PF3*). The results obtained showed that the GA provided the most accurate and balanced results in terms of sensitivity between all the classes using the proposed evaluation metric (*FoM_Penalized_*), increasing the median tumor sensitivity by ~21% with respect to the second best approach and by ~30% with respect to the reference results obtained with 128 bands. After identifying the most relevant bands for each HS test image, the coincident and non-coincident bands were evaluated. The quantitative and qualitative results showed that the selected bands (48 in total that involved the coincident and non-coincident bands) offered similar and even better results in some cases than the reference results obtained with 128 bands. Therefore, for this particular brain cancer detection application, the most relevant spectral ranges identified were: 440.5–465.96 nm, 498.71–509.62 nm, 556.91–575.1 nm, 593.29–615.12 nm, 636.94–666.05 nm, 698.79–731.53 nm and 884.32–902.51 nm.

Although this preliminary study achieved a reduction of the information captured by the HS sensor within the spectral range between 400 to 1000 nm, the rationale of this experiment was to try to identify the most relevant wavelengths and provide an accurate differentiation of the tissue types and materials presented in a neurosurgical scene. As has been demonstrated in this work, the use of the entire number of spectral bands captured by the HS sensor (826) is unnecessary to achieve an accurate classification. Furthermore, the proposed reduction in the number of bands can remove spurious spectral information that produces misclassifications between the different classes, especially between the tumor class and the other classes. As has been presented in the quantitative and qualitative results, no loss of detail is obtained within the proposed methodology.

Due to the challenges of obtaining good-quality HS data during surgical procedures, especially in brain surgery, using the intraoperative macroscopic HS acquisition system based on push-broom HS cameras, the number of patients currently included in the HS brain cancer database is not high enough to state that the proposed method is robust and general. The work presented in this paper is a preliminary study where we demonstrate, as a proof-of-concept, that using a reduced number of wavelengths the accuracy of the results remains constant with respect to the employment of the original number of wavelengths. Future works will be focused in the inclusion of more patients in the training and test datasets in order to validate the spectral ranges preliminarily identified in this study as the most relevant for this application. The use of animal studies with a large number of subjects could be considered as a complement to strongly validate the proposed methodology. In addition, the use of an improved methodology to select the final coincident and non-coincident bands with the goal of reducing as much as possible the number of bands, preserving the accuracy of the results, will be explored.

Further experiments will be carried out to improve the classification results achieved with the reduced number of bands by including the spatial information in conjunction with the spectral information. The inclusion of the spatial relationship among pixels could lead to a reduction of the false positives/negatives in the classification results, achieving better delineation of the tumor areas. Nevertheless, the inclusion of the spatial information is out of the scope of this research, where we are targeting identification of the most relevant spectral features, which will allow a cost reduction in the instrumentation and in the time required to train a classifier. Moreover, future works will explore the use of deep learning approaches to improve the classification results using more data but with a reduced number of bands to evaluate if deep-learning methods outperform traditional SVM-based approaches when the number of spectral bands is extremely reduced.

In this preliminary study, the evaluation of the tumor margin delineation provided by the proposed algorithm was performed through visual inspection of the classification results by the operating surgeon, due to the impossibility of performing a pathological assessment of the entire brain tissue sample. This limitation should be addressed in future studies in order to confirm the validity of the results. A possible approach for this validation could be performing several biopsies, during the surgical procedure, in different points of the tumor area (especially in the margins) that will be intraoperatively identified by the system. Then, the histopathological analysis of such samples will be carried out in order to confirm the accuracy of the results obtained by the classification algorithm. In addition, further experiments should be accomplished in controlled environments with the goal of establishing a correlation between the selected wavelengths and the biological properties of the tissue, especially the contributions of hemoglobin and water for the tissue-type differentiation. Moreover, a preliminary segmentation of the parenchymal area and an accurate identification of the blood vessels’ map of the exposed brain, performed before classification, could improve the results in the discrimination of the normal and tumor tissues using a binary classifier specifically trained to identify the relevant biological differences between these two tissue types.

The methodologies proposed in this preliminary study could be extrapolated to intraoperatively analyze other types of cancers in other organs using HSI. Several studies have been performed in the literature to analyze the use of HSI for cancer detection in different tissue types [4]. In this sense, the use of the proposed methodology could be applied to these databases in order to find the most relevant wavelengths for each particular application.

Finally, the results obtained with the proposed methodology based on the GA optimization (PF2-GA-FoM_P in *L1*) demonstrated that using only 48 bands, the quantitative classification results for the tumor class identification are slightly improved in ~5% with respect to the reference results employing the 128 bands. Although this result is not highly significant, especially taking into account the high standard deviations, it is worth noticing that the use of a reduced number of bands for the acquisition will accelerate both the acquisition time (customized HS sensors could be developed to provide real-time HS imaging) and the processing time of the data. The results of our experiments motivate the use of simpler HS cameras for the acquisition of intraoperative brain images, reducing the complexity of the instrumentation and enabling the possibility of its integration in surgical microscopes. In addition, this will motivate the use of snapshot HS cameras with an optimized spectral range tuned to this application, which would make possible the acquisition of intraoperative HS video during surgical procedures. In this sense, the number of data available for training machine-learning models would increase, and thus, the classification would be further improved. The enhancement of the HS database will be mandatory to fulfill our long-term goal that focuses on providing hospitals all over the world with a new generation of aid-visualization systems based on HSI technology that could help surgeons in routine clinical practice.

## Figures and Tables

**Table 1:** Summary of the hyperspectral (HS)-labeled dataset employed in this study.

Class	#Labeled Pixels	#Images	#Patients
Normal Tissue	101,706	26	16
Tumor Tissue (Glioblastoma-GBM)	11,054	6	4
Hypervascularized Tissue	38,784	25	16
Background	118,132	24	15
Total	269,676	26	16

**Table 2:** Summary of the HS dataset with different sampling intervals and number of spectral bands.

	#Spectral Bands										
	826	645	320	214	160	128	80	64	32	16	8
**λmin (nm)**	400	440	440	440	440	440	440	440	440	440	440
**λmax (nm)**	1000	902	902	902	902	902	902	902	902	902	902
**Sampling Interval (nm)**	0.73	0.73	1.44	2.16	2.89	3.61	5.78	7.22	14.44	28.88	57.75
**Size (MB)**	1328.3	1037.3	514.6	344.1	257.3	205.8	128.6	102.9	51.4	25.7	12.8

**Table 3:** Average and standard deviation (STD) of overall accuracy (OA), Matthews correlation coefficient (MCC), sensitivity and specificity of all images.

Level (#bands)	OA (%) (STD)	MCC (%) (STD)	Sensitivity (%) - (STD)				Specificity (%) - (STD)			
			NT	TT	HT	BG	NT	TT	HT	BG
**L1 (48)**	77.9 (17.0)	83.6 (9.1)	85.1 (17.6)	52.7 (29.8)	83.5 (20.9)	92.5 (14.2)	87.3 (12.2)	94.6 (8.3)	96.7 (5.1)	85.3 (18.0)
**L2 (22)**	77.0 (16.8)	83.3 (8.6)	83.7 (19.9)	57.0 (32.6)	81.9 (23.0)	90.1 (20.1)	85.2 (13.4)	91.2 (14.4)	97.1 (4.9)	87.7 (17.6)
**L3 (2)**	53.8 (21.2)	68.9 (11.4)	52.8 (42.6)	57.6 (36.5)	48.8 (26.4)	84.8 (27.1)	72.9 (13.2)	70.3 (30.8)	93.1 (8.0)	80.0 (21.1)

NT: Normal Tissue; TT: Tumor Tissue; HT: Hypervascularized Tissue; BG: Background.

## Supplementary Materials

The following are available online at https://www.mdpi.com/1424-8220/19/24/5481/s1: Table S1. Summary of the labeled dataset employed in the experiments. The numbering of the data corresponds with the dataset publically available in [47]; Figure S1. Average and standard deviation results of the leave-one-patient-out cross-validation for each band reduction with different sampling interval values. (a) Using the original training dataset. (b) Using the reduced training dataset; Figure S2. Average and standard deviation results of the normalized MCC metric using leave-one-patient-out cross-validation for each band reduction with different sampling interval values. (a) Using the original training dataset. (b) Using the reduced training dataset; Figure S3. Boxplot results of the leave-one-patient-out cross-validation obtained for the *PF3* employing the *ACO* algorithm with different numbers of bands (20, 40, 60, 80 and 100). (a) Overall accuracy. (b) Tumor tissue sensitivity. (c) Normal tissue sensitivity. (d) Hypervascularized tissue sensitivity; Figure S4. Boxplot results of the normalized MCC metric using leave-one-patient-out cross-validation obtained for the *PF3* employing the *ACO* algorithm with different numbers of bands (20, 40, 60, 80 and 100). (a) Tumor tissue. (b) Normal tissue. (c) Hypervascularized tissue; Table S2. Identification of the specific 48 wavelengths selected by the *GA* in the *PF2* using the *FoM_Penalized_* evaluation metric and the *L1* band combination.

## Appendix A

**Table A1:** Acronym list of the proposed methods employed in this study.

**PF1-128**	Processing Framework 1 using 128 bands
**PF2-GA-OA_P**	Processing Framework 2 using Genetic Algorithm and the Overall Accuracy Penalized evaluation metric
**PF2-GA-FoM_P**	Processing Framework 2 using Genetic Algorithm and the Figure of Merit Penalized evaluation metric
**PF2-PSO-OA_P**	Processing Framework 2 using Particle Swarm Optimization algorithm and the Overall Accuracy Penalized evaluation metric
**PF2-PSO-FoM_P**	Processing Framework 2 using Particle Swarm Optimization algorithm and the Figure of Merit Penalized evaluation metric
**PF3-ACO-60**	Processing Framework 3 using Ant Colony Optimization algorithm taking into account 60 bands

## References

[1] Hammill K., Stewart C.G., Kosic N., Bellamy L., Irvine H., Hutley D., Arblaster K. (2019). Exploring the impact of brain cancer on people and their participation. Br. J. Occup. Ther..

[2] Joshi D.M., Rana N.K., Misra V.M. (2010). Classification of Brain Cancer using Artificial Neural Network. Proceedings of the 2010 2nd International Conference on Electronic Computer Technology.

[3] Perkins A., Liu G. (2016). Primary Brain Tumors in Adults: Diagnosis and Treatment—American Family Physician. Am. Fam. Physician.

[4] Halicek M., Fabelo H., Ortega S., Callico G.M., Fei B. (2019). In-Vivo and Ex-Vivo Tissue Analysis through Hyperspectral Imaging Techniques: Revealing the Invisible Features of Cancer. Cancers.

[5] Kamruzzaman M., Sun D.W. (2016). Introduction to Hyperspectral Imaging Technology. Comput. Vis. Technol. Food Qual. Eval..

[6] Mordant D.J., Al-Abboud I., Muyo G., Gorman A., Sallam A., Ritchie P., Harvey A.R., McNaught A.I. (2011). Spectral imaging of the retina. Eye.

[7] Johnson W.R., Wilson D.W., Fink W., Humayun M., Bearman G. (2007). Snapshot hyperspectral imaging in ophthalmology. J. Biomed. Opt..

[8] Gao L., Smith R.T., Tkaczyk T.S. (2012). Snapshot hyperspectral retinal camera with the Image Mapping Spectrometer (IMS). Biomed. Opt. Express.

[9] Akbari H., Kosugi Y., Kojima K., Tanaka N. (2010). Detection and Analysis of the Intestinal Ischemia Using Visible and Invisible Hyperspectral Imaging. IEEE Trans. Biomed. Eng..

[10] Ortega S., Fabelo H., Camacho R., Plaza M.L., Callicó G.M., Sarmiento R. (2018). Detecting brain tumor in pathological slides using hyperspectral imaging. Biomed. Opt. Express.

[11] Zhu S., Su K., Liu Y., Yin H., Li Z., Huang F., Chen Z., Chen W., Zhang G., Chen Y. (2015). Identification of cancerous gastric cells based on common features extracted from hyperspectral microscopic images. Biomed. Opt. Express.

[12] Lu C., Mandal M. (2014). Toward automatic mitotic cell detection and segmentation in multispectral histopathological images. IEEE J. Biomed. Health Inform..

[13] Khouj Y., Dawson J., Coad J., Vona-Davis L. (2018). Hyperspectral Imaging and K-Means Classification for Histologic Evaluation of Ductal Carcinoma In Situ. Front. Oncol..

[14] Bjorgan A., Denstedt M., Milanič M., Paluchowski L.A., Randeberg L.L., Alfano R.R., Demos S.G. (2015). Vessel contrast enhancement in hyperspectral images. Optical Biopsy XIII: Toward Real-Time Spectroscopic Imaging and Diagnosis.

[15] Akbari H., Kosugi Y., Kojima K., Tanaka N. Blood vessel detection and artery-vein differentiation using hyperspectral imaging. Proceedings of the 31st Annual International Conference of the IEEE Engineering in Medicine and Biology Society: Engineering the Future of Biomedicine, EMBC 2009.

[16] Milanic M., Bjorgan A., Larsson M., Strömberg T., Randeberg L.L., Brown J.Q., Deckert V. (2015). Detection of hypercholesterolemia using hyperspectral imaging of human skin. Proceedings of the SPIE—European Conference on Biomedical Optics.

[17] Zhi L., Zhang D., Yan J., Li Q.L., Tang Q. (2007). Classification of hyperspectral medical tongue images for tongue diagnosis. Comput. Med. Imaging Graph..

[18] Yudovsky D., Nouvong A., Pilon L. (2010). Hyperspectral Imaging in Diabetic Foot Wound Care. J. Diabetes Sci. Technol..

[19] Lu G., Fei B. (2014). Medical hyperspectral imaging: A review. J. Biomed. Opt..

[20] Calin M.A., Parasca S.V., Savastru D., Manea D. (2014). Hyperspectral imaging in the medical field: Present and future. Appl. Spectrosc. Rev..

[21] Ortega S., Fabelo H., Iakovidis D., Koulaouzidis A., Callico G., Ortega S., Fabelo H., Iakovidis D.K., Koulaouzidis A., Callico G.M. (2019). Use of Hyperspectral/Multispectral Imaging in Gastroenterology. Shedding Some–Different–Light into the Dark. J. Clin. Med..

[22] Akbari H., Uto K., Kosugi Y., Kojima K., Tanaka N. (2011). Cancer detection using infrared hyperspectral imaging. Cancer Sci..

[23] Kiyotoki S., Nishikawa J., Okamoto T., Hamabe K., Saito M., Goto A., Fujita Y., Hamamoto Y., Takeuchi Y., Satori S. (2013). New method for detection of gastric cancer by hyperspectral imaging: A pilot study. J. Biomed. Opt..

[24] Baltussen E.J.M., Kok E.N.D., Brouwer de Koning S.G., Sanders J., Aalbers A.G.J., Kok N.F.M., Beets G.L., Flohil C.C., Bruin S.C., Kuhlmann K.F.D. (2019). Hyperspectral imaging for tissue classification, a way toward smart laparoscopic colorectal surgery. J. Biomed. Opt..

[25] Han Z., Zhang A., Wang X., Sun Z., Wang M.D., Xie T. (2016). In vivo use of hyperspectral imaging to develop a noncontact endoscopic diagnosis support system for malignant colorectal tumors. J. Biomed. Opt..

[26] Panasyuk S.V., Yang S., Faller D.V., Ngo D., Lew R.A., Freeman J.E., Rogers A.E. (2007). Medical hyperspectral imaging to facilitate residual tumor identification during surgery. Cancer Biol. Ther..

[27] Pourreza-Shahri R., Saki F., Kehtarnavaz N., Leboulluec P., Liu H. Classification of ex-vivo breast cancer positive margins measured by hyperspectral imaging. Proceedings of the 2013 IEEE International Conference on Image Processing, ICIP 2013.

[28] Lu G., Halig L., Wang D., Chen Z.G., Fei B., Yaniv Z.R., Holmes D.R. (2014). Hyperspectral imaging for cancer surgical margin delineation: Registration of hyperspectral and histological images. SPIE Medical Imaging 2014: Image-Guided Procedures, Robotic Interventions, and Modeling.

[29] Pike R., Lu G., Wang D., Chen Z.G., Fei B. (2016). A Minimum Spanning Forest-Based Method for Noninvasive Cancer Detection With Hyperspectral Imaging. IEEE Trans. Biomed. Eng..

[30] Fei B., Lu G., Wang X., Zhang H., Little J.V., Patel M.R., Griffith C.C., El-Diery M.W., Chen A.Y. (2017). Label-free reflectance hyperspectral imaging for tumor margin assessment: A pilot study on surgical specimens of cancer patients. J. Biomed. Opt..

[31] Halicek M., Little J.V., Wang X., Chen A.Y., Fei B. (2019). Optical biopsy of head and neck cancer using hyperspectral imaging and convolutional neural networks. J. Biomed. Opt..

[32] Regeling B., Thies B., Gerstner A.O.H., Westermann S., Müller N.A., Bendix J., Laffers W. (2016). Hyperspectral Imaging Using Flexible Endoscopy for Laryngeal Cancer Detection. Sensors.

[33] Halicek M., Dormer J.D., Little J.V., Chen A.Y., Myers L., Sumer B.D., Fei B. (2019). Hyperspectral Imaging of Head and Neck Squamous Cell Carcinoma for Cancer Margin Detection in Surgical Specimens from 102 Patients Using Deep Learning. Cancers.

[34] Fabelo H., Ortega S., Ravi D., Kiran B.R., Sosa C., Bulters D., Callicó G.M., Bulstrode H., Szolna A., Piñeiro J.F. (2018). Spatio-spectral classification of hyperspectral images for brain cancer detection during surgical operations. PLoS ONE.

[35] Fabelo H., Ortega S., Lazcano R., Madroñal D., M Callicó G., Juárez E., Salvador R., Bulters D., Bulstrode H., Szolna A. (2018). An Intraoperative Visualization System Using Hyperspectral Imaging to Aid in Brain Tumor Delineation. Sensors.

[36] Fabelo H., Halicek M., Ortega S., Shahedi M., Szolna A., Piñeiro J.F., Sosa C., O’Shanahan A.J., Bisshopp S., Espino C. (2019). Deep Learning-Based Framework for In Vivo Identification of Glioblastoma Tumor using Hyperspectral Images of Human Brain. Sensors.

[37] Ghamisi P., Plaza J., Chen Y., Li J., Plaza A.J. (2017). Advanced Spectral Classifiers for Hyperspectral Images: A review. IEEE Geosci. Remote Sens. Mag..

[38] Van Der Maaten L.J.P., Postma E.O., Van Den Herik H.J. (2009). Dimensionality Reduction: A Comparative Review. J. Mach. Learn. Res..

[39] Lunga D., Prasad S., Crawford M.M., Ersoy O. (2014). Manifold-Learning-Based Feature Extraction for Classification of Hyperspectral Data: A Review of Advances in Manifold Learning. IEEE Signal Process. Mag..

[40] Dai Q., Cheng J.H., Sun D.W., Zeng X.A. (2015). Advances in Feature Selection Methods for Hyperspectral Image Processing in Food Industry Applications: A Review. Crit. Rev. Food Sci. Nutr..

[41] Sastry K., Goldberg D.E., Kendall G. (2014). Genetic Algorithms. Search Methodologies.

[42] Perez R.E., Behdinan K. (2012). Particle Swarm Optimization in Structural Design. Swarm Intell. Focus Ant Part. Swarm Optim..

[43] Sharma S., Buddhiraju K.M. (2018). Spatial-spectral ant colony optimization for hyperspectral image classification. Int. J. Remote Sens..

[44] Rashmi S., Addamani S., Ravikiran A. (2014). Spectral Angle Mapper algorithm for remote sensing image classification. IJISET Int. J. Innov. Sci. Eng. Technol..

[45] Halicek M., Fabelo H., Ortega S., Little J.V., Wang X., Chen A.Y., Callicó G.M., Myers L., Sumer B., Fei B., Fei B., Linte C.A. (2019). Cancer detection using hyperspectral imaging and evaluation of the superficial tumor margin variance with depth. Medical Imaging 2019: Image-Guided Procedures, Robotic Interventions, and Modeling.

[46] Lu G., Qin X., Wang D., Chen Z.G., Fei B. Quantitative wavelength analysis and image classification for intraoperative cancer diagnosis with hyperspectral imaging. Proceedings of the Progress in Biomedical Optics and Imaging—Proceedings of SPIE.

[47] Fabelo H., Ortega S., Szolna A., Bulters D., Pineiro J.F., Kabwama S., J-O’Shanahan A., Bulstrode H., Bisshopp S., Kiran B.R. (2019). In-Vivo Hyperspectral Human Brain Image Database for Brain Cancer Detection. IEEE Access.

[48] Chen P.C., Lin W.C. (2011). Spectral-profile-based algorithm for hemoglobin oxygen saturation determination from diffuse reflectance spectra. Biomed. Opt. Express.

[49] Eaton W.A., Hanson L.K., Stephens P.J., Sutherland J.C., Dunn J.B.R. (1978). Optical spectra of oxy- and deoxyhemoglobin. J. Am. Chem. Soc..

[50] Sekar S.K.V., Bargigia I., Mora A.D., Taroni P., Ruggeri A., Tosi A., Pifferi A., Farina A. (2017). Diffuse optical characterization of collagen absorption from 500 to 1700 nm. J. Biomed. Opt..

[51] Camps-Valls G., Bruzzone L., Electr E., Escola T., Val U. (2005). De Kernel-based methods for hyperspectral image classification. IEEE Trans. Geosci. Remote Sens..

[52] Fabelo H., Halicek M., Ortega S., Szolna A., Morera J., Sarmiento R., Callicó G.M., Fei B. (2019). Surgical aid visualization system for glioblastoma tumor identification based on deep learning and in-vivo hyperspectral images of human patients. Medical Imaging 2019: Image-Guided Procedures, Robotic Interventions, and Modeling.

[53] Chang C.C., Lin C.J. (2011). LIBSVM: A library for support vector machines. ACM Trans. Intell. Syst. Technol..

[54] Moore A. K-means and Hierarchical Clustering. http://www.cs.cmu.edu/afs/cs/user/awm/web/tutorials/kmeans11.pdf.

[55] Akhter N., Dabhade S., Bansod N., Kale K. (2016). Feature Selection for Heart Rate Variability Based Biometric Recognition Using Genetic Algorithm. Intelligent Systems Technologies and Applications.

[56] Haupt S.E., Haupt R.L. (2007). Genetic algorithms and their applications in environmental sciences. Advanced Methods for Decision Making and Risk Management in Sustainability Science.

[57] Zortea M., Plaza A. (2009). Spatial Preprocessing for Endmember Extraction. IEEE Trans. Geosci. Remote Sens..

[58] Boughorbel S., Jarray F., El-Anbari M. (2017). Optimal classifier for imbalanced data using Matthews Correlation Coefficient metric. PLoS ONE.

