# Supplementary material for: Most Relevant Spectral Bands Identification for Brain Cancer Detection Using Hyperspectral Imaging
Source: Sensors (Basel). 2019 Dec 12;19(24):5481. doi: 10.3390/s19245481 (PMC6961052; doi:10.3390/s19245481)
Supplement: Supplementary file 1 [file sensors-19-05481-s001.pdf]

Most Relevant Spectral Bands Identification for Brain Cancer Detection using Hyperspectral Imaging

Supplementary Material

Table S1. Summary of the labeled dataset employed in the experiments. The numbering of the data corresponds with the dataset publically available in [47].

Patient ID	Image ID	Size (height x width x bands)	#Labeled Pixels (#Reference Pixels)				Diagnosis
			NT	TT	HT	BG	
004	02	389 x 345 x 826	5,007	0	965	1,992	Normal Brain
005	01	483 x 488 x 826	6,061	0	1,727	20,483	Normal Brain
007	01	582 x 400 x 826	7,714	0	1,089	0	Normal Brain
008	01	460 x 549 x 826	2,295	1,221	1,331	630	Grade IV Glioblastoma (P)
	02	480 x 553 x 826	2,187	138	1,000	7,444	Grade IV Glioblastoma (P)
010	03	371 x 461 x 826	10,626	0	2,332	3,972	Grade IV Glioblastoma (P)
012	01	443 x 497 x 826	4,516	855	8,697	1,685	Grade IV Glioblastoma (P)
	02	445 x 498 x 826	6,553	3,139	6,041	8,731	Grade IV Glioblastoma (P)
013	01	298 x 253 x 826	1,827	0	129	589	Normal Brain
014	01	317 x 244 x 826	0	0	64	1,866	Grade IV Glioblastoma (P)
015	01	376 x 494 x 826	1,251	2,046	4,089	696	Grade IV Glioblastoma (P)
016	01	335 x 323 x 826	3,970	0	246	12,002	Normal Brain
	02	335 x 326 x 826	349	0	0	2,767	Normal Brain
	03	315 x 321 x 826	603	0	234	1,696	Normal Brain
	04	383 x 297 x 826	1,178	0	1,064	956	Grade IV Glioblastoma (P)
	05	414 x 292 x 826	2,643	0	452	5,125	Grade IV Glioblastoma (P)
017	01	441 x 399 x 826	1,328	0	68	3,069	Grade IV Glioblastoma (P)
018	01	479 x 462 x 826	13,450	0	488	9,773	Normal Brain
	02	510 x 434 x 826	4,813	0	658	5,895	Normal Brain
019	01	601 x 535 x 826	6,499	0	1,350	1,933	Meningioma
020	01	378 x 330 x 826	1,842	3,655	1,513	2,625	Grade IV Glioblastoma (P)
021	01	452 x 334 x 826	2,663	0	2325	5,651	Normal Brain
	02	448 x 324 x 826	2,313	0	591	4,688	Normal Brain
	05	433 x 340 x 826	1038	0	587	10,187	Normal Brain
022	01	597 x 527 x 826	2,806	0	1,064	3,677	Normal Brain
	02	611 x 527 x 826	8,174	0	680	0	Normal Brain
Total: 16 Operations - 26 Captures			101,706	11,054	38,784	118,132	

* NT: Normal Tissue; TT: Tumor Tissue; HT: Hypervascularized Tissue; BG: Background.

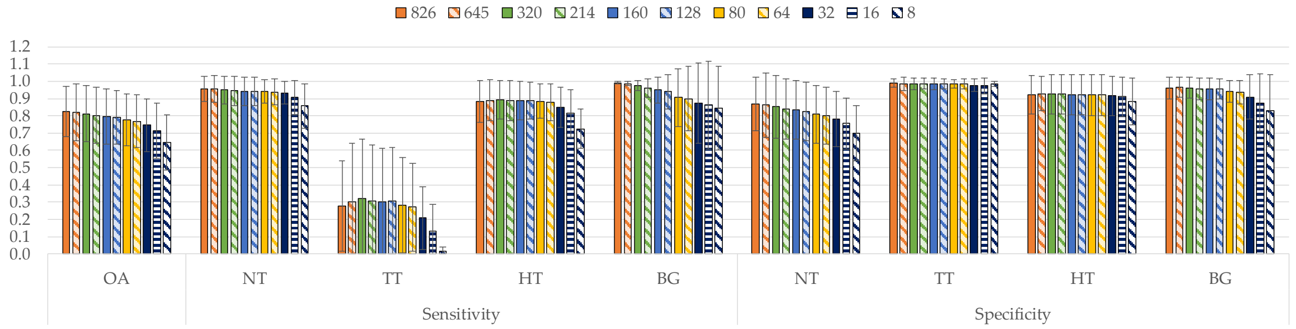

(**a**)

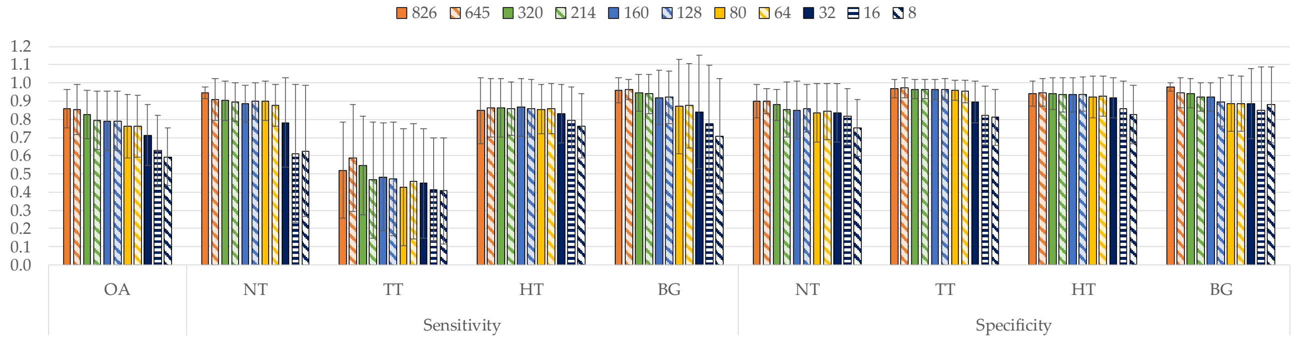

(**b**)

Figure S1. Average and standard deviation results of the leave-one-patient-out cross-validation for each band reduction with different sampling interval values. (**a**) Using the original training dataset. (**b**) Using the reduced training dataset.

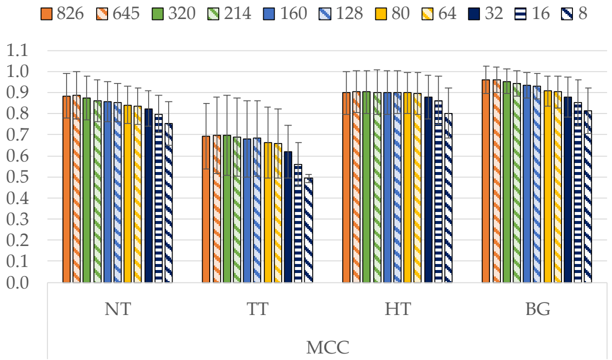

(**a**)

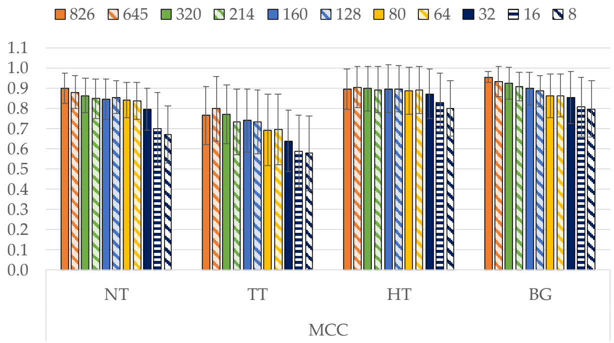

(**b**)

Figure S2. Average and standard deviation results of the normalized MCC metric using leave-one-patient-out cross-validation for each band reduction with different sampling interval values. (**a**) Using the original training dataset. (**b**) Using the reduced training dataset.

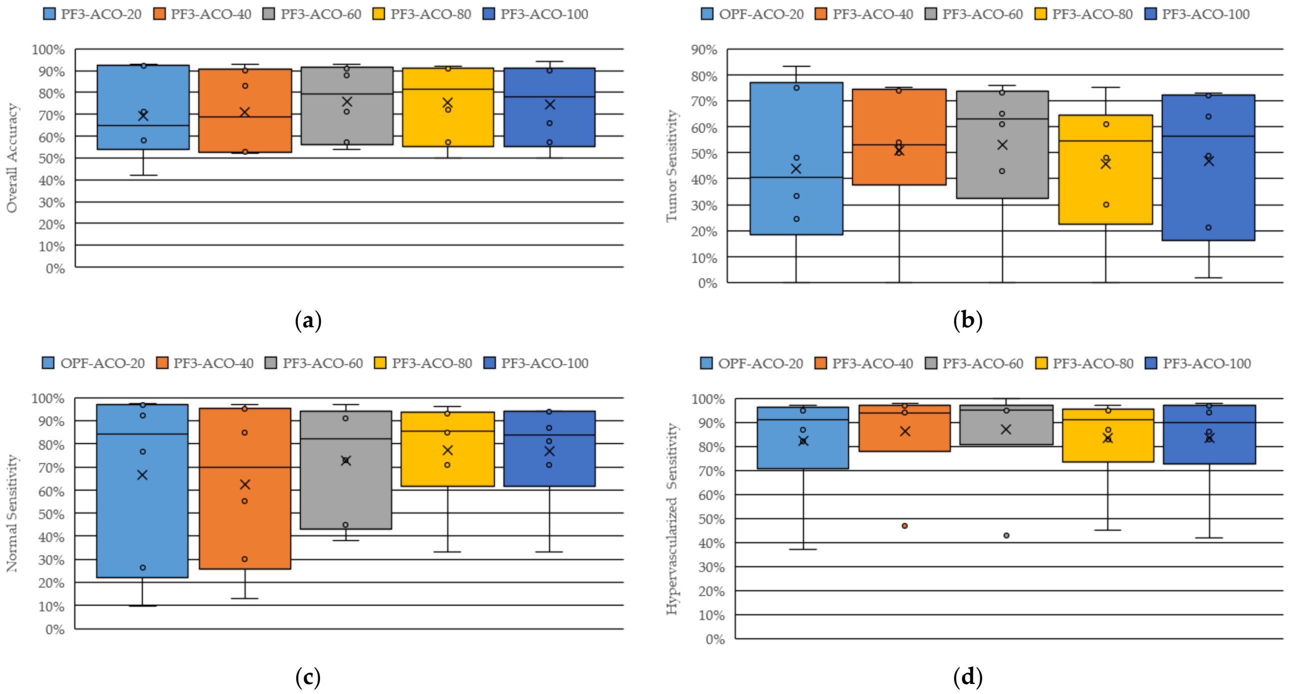

Figure S3. Boxplot results of the leave-one-patient-out cross-validation obtained for the *PF3* employing the *ACO* algorithm with different number of bands (20, 40, 60, 80 and 100). (**a**) Overall accuracy. (**b**) Tumor tissue sensitivity. (**c**) Normal tissue sensitivity. (**d**) Hypervascularized tissue sensitivity.

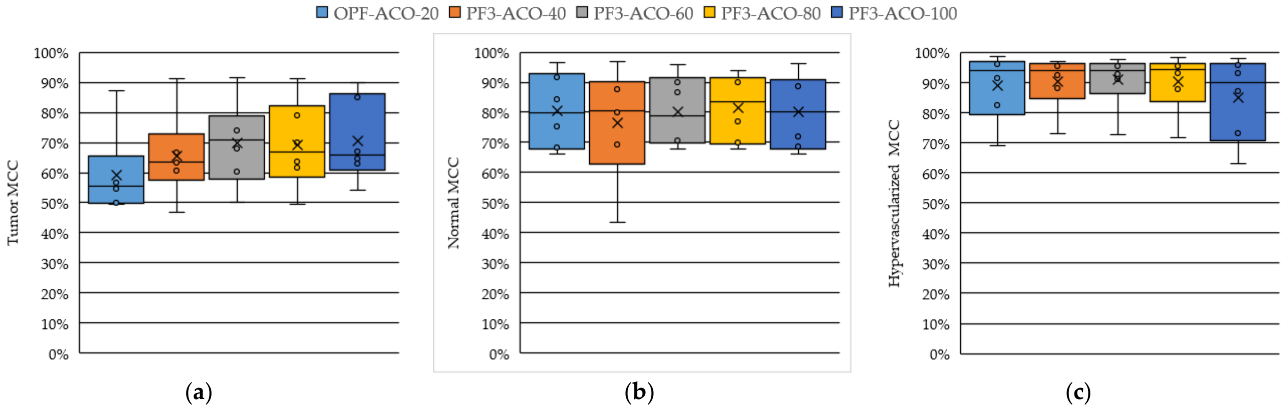

Figure S4. Boxplot results of the normalized MCC metric using leave-one-patient-out cross-validation obtained for the *PF3* employing the *ACO* algorithm with different number of bands (20, 40, 60, 80 and 100). (**a**) Tumor tissue. (**b**) Normal tissue. (**c**) Hypervascularized tissue.

Table S2. Identification of the specific 48 wavelengths selected by the *GA* in the *PF2* using the $FoM_{Penalized}$ evaluation metric and the *L1* band combination.

Band ID	λ (nm)	Band ID	λ (nm)	Band ID	λ (nm)	Band ID	λ (nm)	Band ID	λ (nm)	Band ID	λ (nm)
**1**	440.50	**18**	502.34	**43**	593.29	**56**	640.58	**72**	698.79	**95**	782.46
**2**	444.14	**19**	505.98	**44**	596.93	**57**	644.22	**74**	706.06	**101**	804.29
**3**	447.78	**20**	509.62	**45**	600.57	**58**	647.86	**75**	709.70	**104**	815.20
**5**	455.05	**33**	556.91	**46**	604.20	**59**	651.50	**76**	713.34	**117**	862.49
**7**	462.33	**34**	560.55	**47**	607.84	**60**	655.13	**77**	716.98	**123**	884.32
**8**	465.96	**36**	567.83	**49**	615.12	**61**	658.77	**78**	720.62	**124**	887.96
**14**	487.79	**38**	575.10	**53**	629.67	**62**	662.41	**79**	724.25	**126**	895.23
**17**	498.71	**41**	586.01	**55**	636.94	**63**	666.05	**81**	731.53	**128**	902.51
